# On the Trail of the German Purity Law: Distinguishing the Metabolic Signatures of Wheat, Corn and Rice in Beer

**DOI:** 10.3389/fchem.2021.715372

**Published:** 2021-07-20

**Authors:** Stefan A. Pieczonka, Sophia Paravicini, Michael Rychlik, Philippe Schmitt-Kopplin

**Affiliations:** ^1^ Chair of Analytical Food Chemistry, Technical University of Munich, Freising, Germany; ^2^ Research Unit Analytical BioGeoChemistry, Helmholtz Zentrum München, Neuherberg, Germany

**Keywords:** beer, purity law, authentication, metabolomics, foodomics, molecular networking, mass difference network, FTICR-MS

## Abstract

Here, we report a non-targeted analytical approach to investigate the influence of different starch sources on the metabolic signature in the final beer product. An extensive sample set of commercial beers brewed with barley, wheat, corn and/or rice were analyzed by both direct infusion Fourier transform ion cyclotron mass spectrometry (DI-FTICR MS, 400 samples) and UPLC-ToF-MS (100 samples). By its unrivaled mass resolution and accuracy, DI-FTICR-MS was able to uncover the compositional space of both polar and non-polar metabolites that can be traced back to the use of different starch sources. Reversed phase UPLC-ToF-MS was used to access information about molecular structures (MS^2^-fragmentation spectra) and isomeric separation, with a focus on less polar compounds. Both analytical approaches were able to achieve a clear statistical differentiation (OPLS-DA) of beer samples and reveal metabolic profiles according to the starch source. A mass difference network analysis, applied to the exact marker masses resolved by FTICR, showed a network of potential secondary metabolites specific to wheat, corn and rice. By MS^2^-similarity networks, database and literature search, we were able to identify metabolites and compound classes significant for the use of the different starch sources. Those were also found in the corresponding brewing raw materials, confirming the potential of our approach for quality control and monitoring. Our results also include the identification of the aspartic acid-conjugate of N-β-D-glucopyranosyl-indole-3-acetic acid as a potential marker for the use of rice in the brewing industry regarding quality control and food inspection purposes.

## Introduction

Beer is defined as a fermented, but not distilled, beverage that is made from starch sources. Seen as one of the first food laws, the Bavarian Purity Law (Duke Wilhelm, 1516) stipulates only the ingredients barley, hops and water to ensure the quality standard of beers, its shelf-life, preservation and safety. Nowadays, beverages that are sold as beer are open to a large number of brewing types and raw materials (EuGH, 1987). As an example, the German feudal purity law of 1516 does not allow wheat as an ingredient of beer, because valuable wheat and rye grain should be exclusively used for baking. This contrasts with today’s Bavarian wheat beer, which according to current law (German Beer Purity Law, *Vorläufiges Biergesetz*) must be brewed from at least 50% of wheat malt and top fermented. With Belgian wit beers as a second classic wheat-based beer and especially the rising market of alcohol free beers, the role of wheat and wheat malt significantly changed during the last centuries (Faltermeier et al., 2014).

Other malted grains that are not mentioned in the purity law of 1516 and traditionally used for beer brewing on the international landscape are corn and rice. Rice beers, locally referred to as *zutho*, are found in the Indian cultural area (Teramoto et al., 2002). In the production of gluten-free beer, rice plays a special role as a naturally safe source of starch. Ceccaroni et al. (2019) optimized the malting process of rice for a top fermented gluten-free beer available to individuals affected by celiac disease. By adding caramelized specialty rice malt, a malted and rich aroma profile and an amber color of the rice-only beer was achieved, as the authors report (Mayer et al., 2014). Mayer et al. (2016) claimed to overcome the problems when beer is brewed with rice malt only and reported a bottom-fermenting brewing process based on rice-endogenous enzyme activities. Bailly et al. (2014) report brewing with malted corn in a laboratorial scale, significantly rising the enzyme activity compared to unmalted corn (Santana et al., 2010).

Brewing with corn and rice is therefore diverse, but comes with well-described disadvantages compared to barley malt (Eneje et al., 2004; Zarnkow and Back, 2005; Zarnkow et al., 2005; Meussdoerffer and Zarnkow, 2009; Hager et al., 2014). It also has a significant impact on the beer’s sensory profile (Esslinger, 2009). For these reasons, brewing with a certain proportion of raw grain adjuncts of rice and corn is much more common. In contemporary brewing industry, barley malt is often partially replaced with adjuncts like corn, rice, starch or sugar. Especially the competitive price (O’Rourke, 1999), but also shortened mashing times and lower mashing temperatures make them valuable in modern industrial brewing. The associated changes in enzyme activities, free amino nitrogen and protein content can be balanced out with exogenous enzymes and extracts (Le Van et al., 2001; Zhuang et al., 2016). The use of raw grain and other adjuncts as an inexpensive alternative to barley or wheat malt is forbidden in Germany.

As early as the 1960s, the analytical determination of the use of raw grain adjuncts was in the focus of brewing research in Germany (Schmitt, 1961). Where at that time the original wort difference, mineral content, total and coagulable nitrogen turned out to be characteristics, the carbon isotope determination of the C4 plant corn was subsequently added (Schmitt et al., 1980). Analysis methods based on immunological concepts often showed weak points due to the big expense necessary, cross-reactions or major changes in the beer ingredients during the brewing process (Wagner et al., 1986; Offizorz et al., 1988). Iimure and Sato (2012) investigated the proteome of beers brewed with barley, rice and corn by 2-D gel electrophoresis combined with MS. Following the proteomics approach, two proteins were determined as corn specific but not relevant for the beer quality and thus not further characterized. Fenz (1991) reported the detection of corn adjuncts in beer by HPLC-UV analysis of the corn-specific oxindole derivative 7-hydroxy-2-oxindole-3-acetic acid and glycerol esters of polyphenols after previous extraction and adsorption chromatography. The method described has been further simplified (Pätzold, 1999) and can be used on corn, but not on rice.

The technical advances in separation methods, detector units and mass analyzers, as well as the further development of data collection and analysis, are showing new perspectives for modern beer analysis (Pieczonka S. A. et al., 2021). The entire molecular diversity of beer can be shown and the influence of different raw materials such as hops and wheat on its metabolome can be captured (Pieczonka et al., 2020). Complex reaction networks during the brewing process can be described (Pieczonka SA. et al., 2021). In our study, we report a comprehensive non-targeted analytical approach involving direct-infusion Fourier-transform ion cyclotron mass spectrometry (DI-FTICR-MS) and UPLC-ToF-MS combined with statistical and network analyses to investigate simultaneously the influences of wheat, corn and rice on the beer’s metabolic signature. The findings could be of great interest with regard to quality control in the brewing industry and foodstuff inspection in the context of the Purity Law.

## Materials and Methods

### Direct Infusion Fourier Transform Ion Cyclotron Mass Spectrometry Measurements and Data Processing

A total of 400 samples of commercially available beers from over 40 different countries were analyzed. The sample set is a cross-section representing all possible combinations of beer styles, fermentation types, raw materials, color impressions and alcohol contents available. Thus, metadata co-varying with the characteristic in focus could be excluded. The samples were purchased at local grocery stores between 2018 and 2020 and stored at −20°C prior preparation for analyses. High-resolution mass spectra were acquired on a Bruker solariX Ion Cyclotron Resonance Fourier Transform Mass Spectrometer (Bruker Daltonics GmbH, Bremen, Germany) equipped with a 12 T superconducting magnet (Magnex Scientific Inc., Yarton, GB) and a APOLO II ESI source (BrukerDaltonics GmbH, Bremen, Germany) operated in negative ionization mode. The sample preparation, measurement and data processing parameters were chosen as reported recently (Pieczonka et al., 2020; Pieczonka SA. et al., 2021). An average mass error of < ±0.15 ppm was reached within and between measurement batches. The resulting 7,700 unambiguous molecular formulae in the CHNOSPCl chemical space that occur in at least five samples were kept for further statistical analysis. An overview of the sample set is given in the Supplementary information (Supplementary Table 1).

### UPLC-ToF Measurements and Data Processing

Solid phase extraction (SPE) was applied to a sub-sample set including 100 beers. The SPE-parameters are given in Supplementary Table 2. The eluate was evaporated to dryness (25 °C, 1 mbar, 3 h, Christ Martin™ RVC 2-25 CD vacuum concentrator) dissolved in the starting conditions of the UPLC-gradient, vortexed and centrifuged (4 min at 14,000 rmp). The supernatant, five times concentrated compared to the initial beer sample, was used for UPLC-ToF-MS ESI-negative analysis on a Shimadzu LCMS-9030 Q-ToF-System (Shimadzu Deutschland GmbH, Duisburg, Germany) in randomized order. The parameters of the chromatography and ToF-measurements are given in Supplementary Table 3. A pooled QC consisting of all measured samples was used for system conditioning and measured after every 10th injection. On this basis, the batch was normalized (compensation for intensity fluctuations) by the LOWESS algorithm. A class QC, including all samples with the same carbohydrate source, was used for each of the barley, wheat, rice and corn classes. Features that occur in at least 33% of all samples belonging to the respective class were kept as potential maker features for statistical analysis. The data processing and extraction of chromatographic features was carried out with the open source MS-DIAL software (Tsugawa et al., 2015) after the export of the raw data to the centroided mzML-format within the LabSolutions™ 5.99 SP2 software (Shimadzu Corp., Kyoto, Japan). The data treatment parameters were optimized and are given in Supplementary Table 4.

To validate the origin of the statistically most significant features, 10 g of respective foodstuff (corn grits, corn flour, corn starch, corn oil, wheat grits, wheat flour, whole-wheat flour, wheat starch, rice grits, rice flour, rice starch) including typical grain adjuncts in the brewing industry, was extracted with 40 ml MeOH for 1 h on the shaker (250 min^−1^). The suspensions were centrifuged (5 min, 14.000 rmp) and the supernatant was evaporated to dryness (25 °C, 1 mbar, 8 h). The residue was resolved in 2 ml starting conditions by vortexing and supersonification and syringe filtrated (0.2 µm) before UPLC-ToF-MS analysis. Furthermore, potential marker substances were measured in positive ESI mode to obtain another complementary fragmentation spectrum.

The aspartic acid conjugate of (*6,6-d*_*2*_) N-β-D-glucopyranosyl-indole-3-acetic acid was synthesized as described by Kai et al. (2007) and kindly provided by the latter authors. The standard was resolved in methanol (6 μg ml^−1^) and added to a worked-up beer sample (sample 325) in equal volumes for co-chromatography.

### Data Treatment and Visualization

We performed a supervised OPLS-DA analysis on both the FTICR and UPLC-ToF dataset-matrices consisting of metabolite features and intensities. Data-pretreatment included zero-filling, data normalization, scaling and transformation (Supplementary Table 4). The Hotelling’s T^2^ test (95%) was applied to prohibit the influence of strong outliers on the models. The lists of the most important masses were defined choosing the highest loadings values. The top characteristic masses were selected within the 95th percentile (385 masses for each carbohydrate source for FTICR and 89 for UPLC-ToF respectively). Potential marker for the use of barley were neglected due to co-varying metadata (Supplementary Figure 1). The goodness of the fit and of the prediction were evaluated with the R^2^ and Q^2^ values. To exclude overfitting, we provide the *p*-value of the Cross-Validation Analysis of Variance (CV-ANOVA). With high values for the quality of prediction (Q^2^) that do not exceed those of the goodness of the fit (R^2^Y) and CV-ANOVA *p*-values far lower than 0.05 for the comparison of between-class against within-class variance, the significance of the models could be confirmed and overfitting excluded (Golbraikh and Tropsha, 2002; Westerhuis et al., 2008). Those elaborations were done in SIMCA 13.0.3.0 (Umetrics, Umeå, Sweden). The statistical parameters of the beer samples (Supplementary Table 1) and OPLS models (Supplementary Table 5) can be found in the Supplementary information. Eight samples with inadequate measurement quality were not integrated into the FTICR-MS statistical model. Three samples were excluded from the models because of information on the ingredient list contrary to their positions in the score plots (FTICR and UPLC-MS). Predicted score values were calculated.

The FTICR-MS marker formulae were depicted in van Krevelen diagrams for each starch source. By plotting H/C versus O/C atomic ratios it is possible to depict common compositional patterns within observations’ markers (Hertkorn et al., 2008; Gougeon et al., 2009; Schmitt-Kopplin et al., 2019). A mass difference network (MDiN) was applied utilizing the NetCalc approach (Tziotis et al., 2011). The nodes, representing the annotated sum formulae, were connected by edges which represent compositional changes corresponding to 250 different (bio)chemical reactions.

The UPLC-ToF marker features were subjected to the open source Cyctoscape software environment (Shannon et al., 2003) to visualize a MS^2^-similarity-network based on similar fragments and neutral losses. The similarity cutoff was set to 0.65. Database search for matching fragmentation spectra was performed using the MS-FINDER (Lai et al., 2018) and MetFrag (Ruttkies et al., 2016) software tools. The entries in the HMDB, FooDB, ChEBI, LipidBlast, LipidMaps, KNApSAcK and PubChem databases were used to carry out a comparison of respective *in silico* fragmentation with our experimental data. The best five hits were examined for their plausibility. When possible, the hits were confirmed through experimental spectra of primary literature. The levels of identification were assigned as suggested by the Metabolomics Standards Initiative (Sumner et al., 2007).

The FTICR-MS and UPLC-ToF-MS data were compared with a mass tolerance of ±5 ppm. Isomeric compounds were merged with the same error tolerance for the UPLC-ToF-MS features. The overlaps were illustrated using pie charts.

## Results

### Direct Infusion Fourier Transform Ion Cyclotron Mass Spectrometry

In the first analytical step, we investigated the metabolome profile of a total of 400 bottled beer samples by direct-infusion Fourier transform ion cyclotron mass spectrometry (DI-FT-ICR-MS) using electrospray (-) ionization. The commercial beer samples covered the numerous facets of beer brewing and included beers manufactured in over 40 countries around the world. By that, we could exclude most co-variating metadata. Despite the abundance of different and combined brewing styles, the craft beer style including the step of dry hopping was found to co-variate with beers brewed with barley only (Supplementary Figure 1).

**FIGURE 1:**
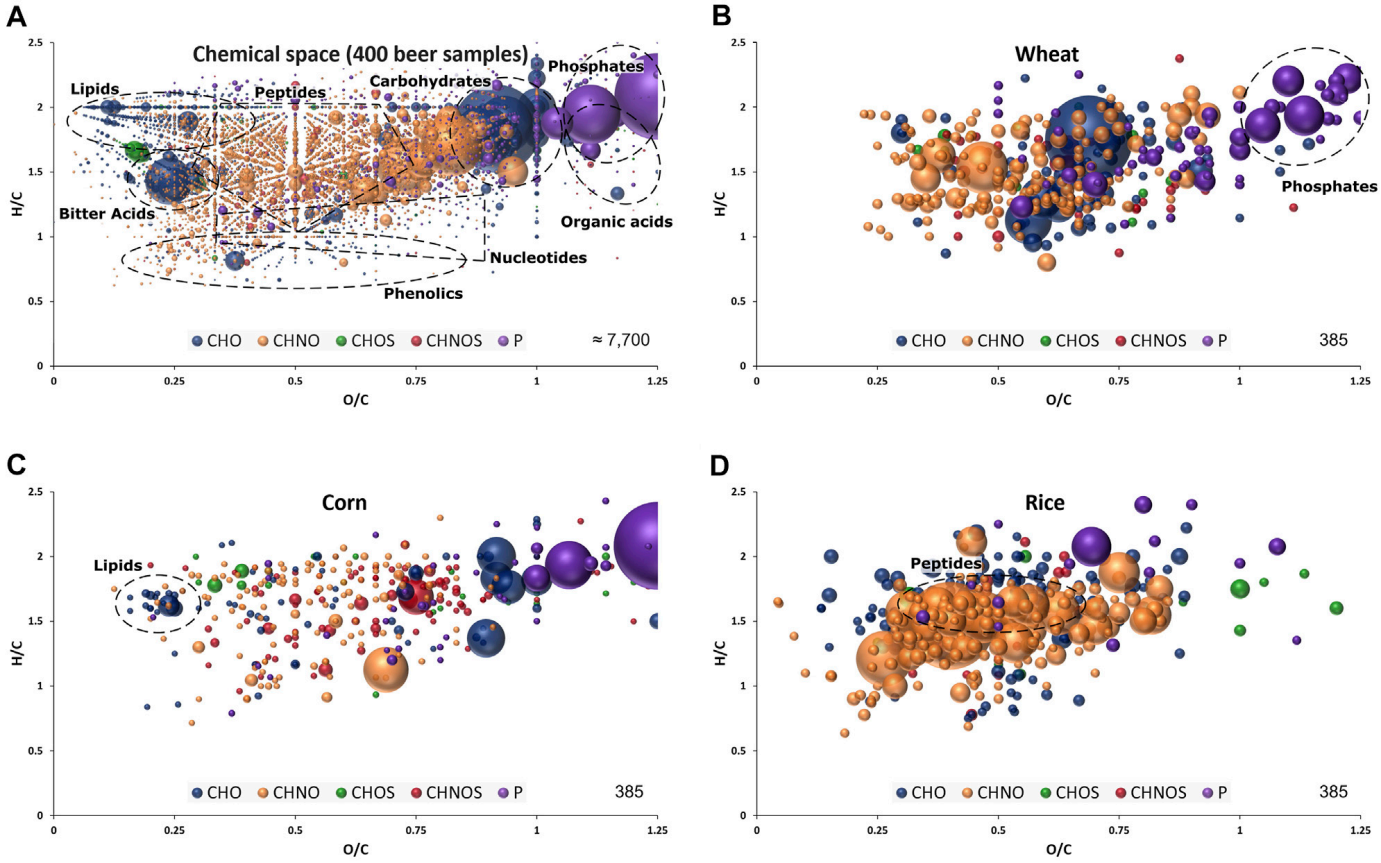
Van Krevelen diagram of molecular formula annotations found in 400 beer samples **(A)** and significant for wheat **(B)**, corn **(C)** and rice **(D)** by FI-FTICR-MS as extracted after OPLS-DA modelling presented in Figure 2. Regions specific to certain compound classes are highlighted. Color code: CHO blue; CHNO orange; CHOS green; CHNOS red; P purple. Neutral molecular formulae are plotted. The bubble size indicates the mean relative intensities of corresponding peaks in the spectra.

The non-targeted and holistic approach, renouncing discriminatory sample processing and chromatography, is capable to resolve the entire molecular complexity of beer within a quick (10 min) measurement. About 7.700 unambiguous molecular compositions could be assigned to exact monoisotopic masses spanning the mass range of *m/z* 100–1,000 (Figure 1A) within the sample batch. They reach from polar sugars, phosphates and sulfates over diverse secondary metabolites and peptides to non-polar lipids, hops bitter acids and highly unsaturated polyphenols and Maillard reaction end products (Pieczonka et al., 2020; Pieczonka SA. et al., 2021). We were able to resolve up to 40 monoisotopic mass features within one single nominal mass including several significant compositions regarding carbohydrate sources, as will be seen later (Supplementary Table 6). The molecular formulae were annotated in the CHNOSP chemical space and subjected to further statistical analysis.

**FIGURE 2:**
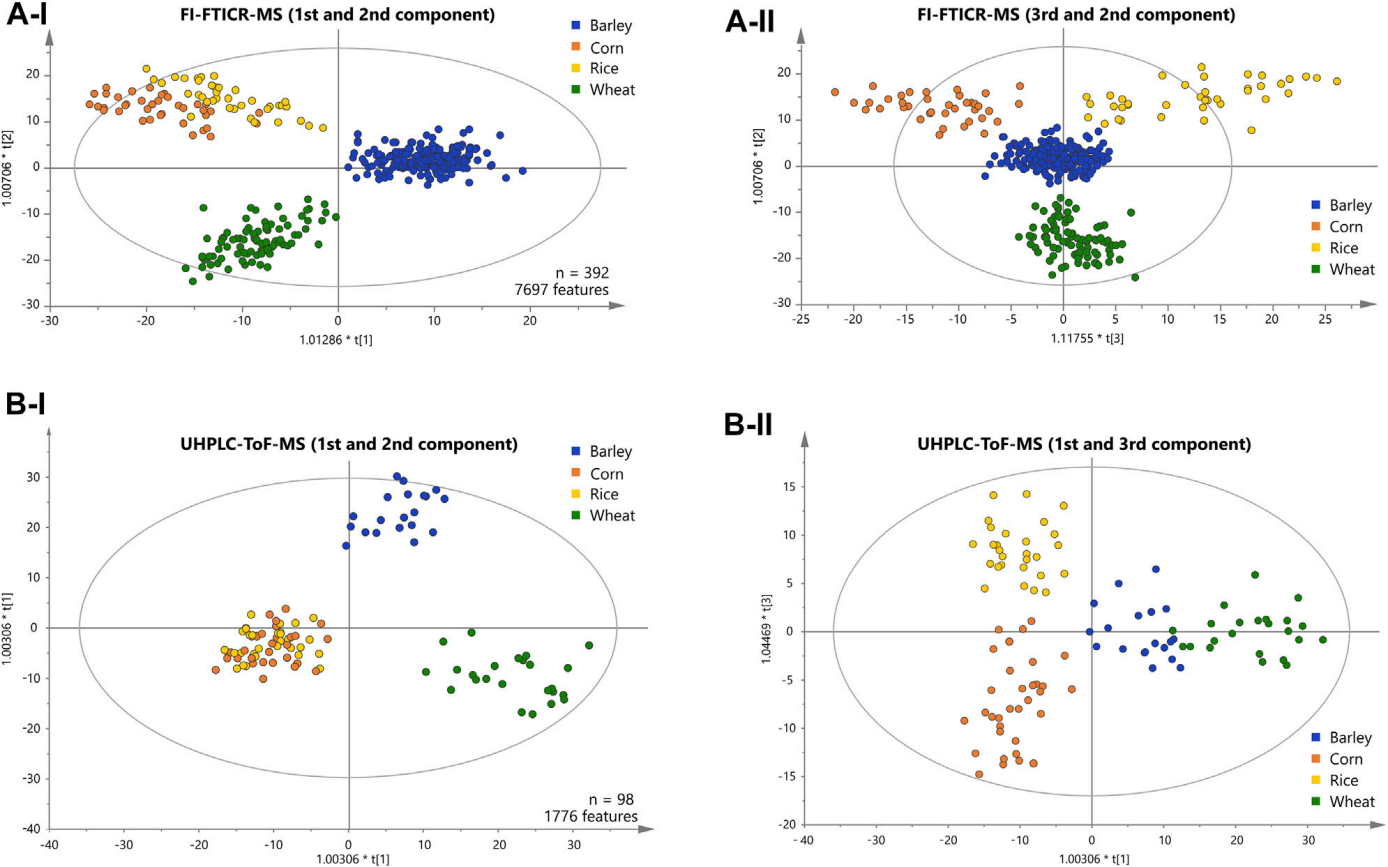
Score plots of the OPLS-DA of the FI-FTICR-MS **(A)** and UPLC-ToF-MS **(B)** data differentiating the carbohydrate sources used. The position of the beer samples is marked by dots colored according to their carbohydrate source. The first and second components are shown in **(A-I)** and **(B-I)**. The third against the second and the first against the third component are shown in **(A-II)** and **(B-II)** respectively.

We applied supervised orthogonal partial least-squares discriminant analysis (OPLS-DA) to the metabolite data resolved by DI-FTICR-MS, using the carbohydrate source as Y-variable. The classification power of the model was highly significant (Supplementary Table 5). The Q^2^ value for quality of prevision (>0.6) and the R2Y value for the goodness of the fit (>0.85) prove the statistical relevance whereas overfitting was excluded by the *p*-value calculated after the CV-ANOVA (<<0.05) (Golbraikh and Tropsha, 2002; Westerhuis et al., 2008). The associated score plot (Figure 2A) showed a clear differentiation of barley, wheat, corn and rice beers. In the first principal component, beers brewed with barley only are separated from beers with an additional carbohydrate source. The second component separates beers brewed with wheat from that brewed with corn or rice (Figure 2A-I). Ultimately, the third component differentiates rice and corn beers (Figure 2A-II). Accordingly, a statistical model was achieved that uncovered the influence of all considered carbohydrate sources on the metabolic signature of beer. Metabolite features that drive the separation were extracted from the respective loadings plot (Supplementary Figure 2). Compositions causing the agglomeration of corn and rice beers in the first and second component are referred to as “corn and rice” features in the following.

The marker metabolites for corn described by Fenz (1991) could be confirmed with significant mass values equal to *p*-coumaroyl glycerol (C_12_H_14_O_5_), caffeoyl glycerol (C_12_H_14_O_6_) and hydroxyoxindoleacetic acid (C_10_H_9_NO_4_). In addition to individual masses, the van Krevelen diagram of the respective compositions revealed characteristic patterns for the carbohydrates sources. Beers brewed with wheat featured a multitude of very polar phosphates (Figure 1B) and beers brewed with corn showed a specific pattern of lipids (Figure 1C). Many compositions characteristic for rice beers are located in the area where peptides are expected (Figure 1D).

Additionally, compositional mass difference networks (MDiN) have proven to be powerful tools to set significant compositions in relation. It can utilize the exact mass information FTICR-MS provides, where compositions are represented as nodes that are connected by edges representing distinct mass differences that describe (bio)chemical relations. Such a MDiN sets in relation the lipid pattern found specific to corn by (de)hydrogenation, hydroxylation, water or glycerol addition and chain elongation reactions (Supplementary Figure 3). Several derivatives of the lipid with the mass *m/z* 335.22278 (C_20_H_32_O_4_) could be explained by e.g., hydrogenation (C_20_H_34_O_4_) and hydroxylation (C_20_H_32_O_5_) reactions. Accordingly, the characteristic composition (C_21_H_34_O_4_) is connected to (C_21_H_36_O_4_) and (C_21_H_34_O_5_) by hydrogenation and hydroxylation respectively.

**FIGURE 3:**
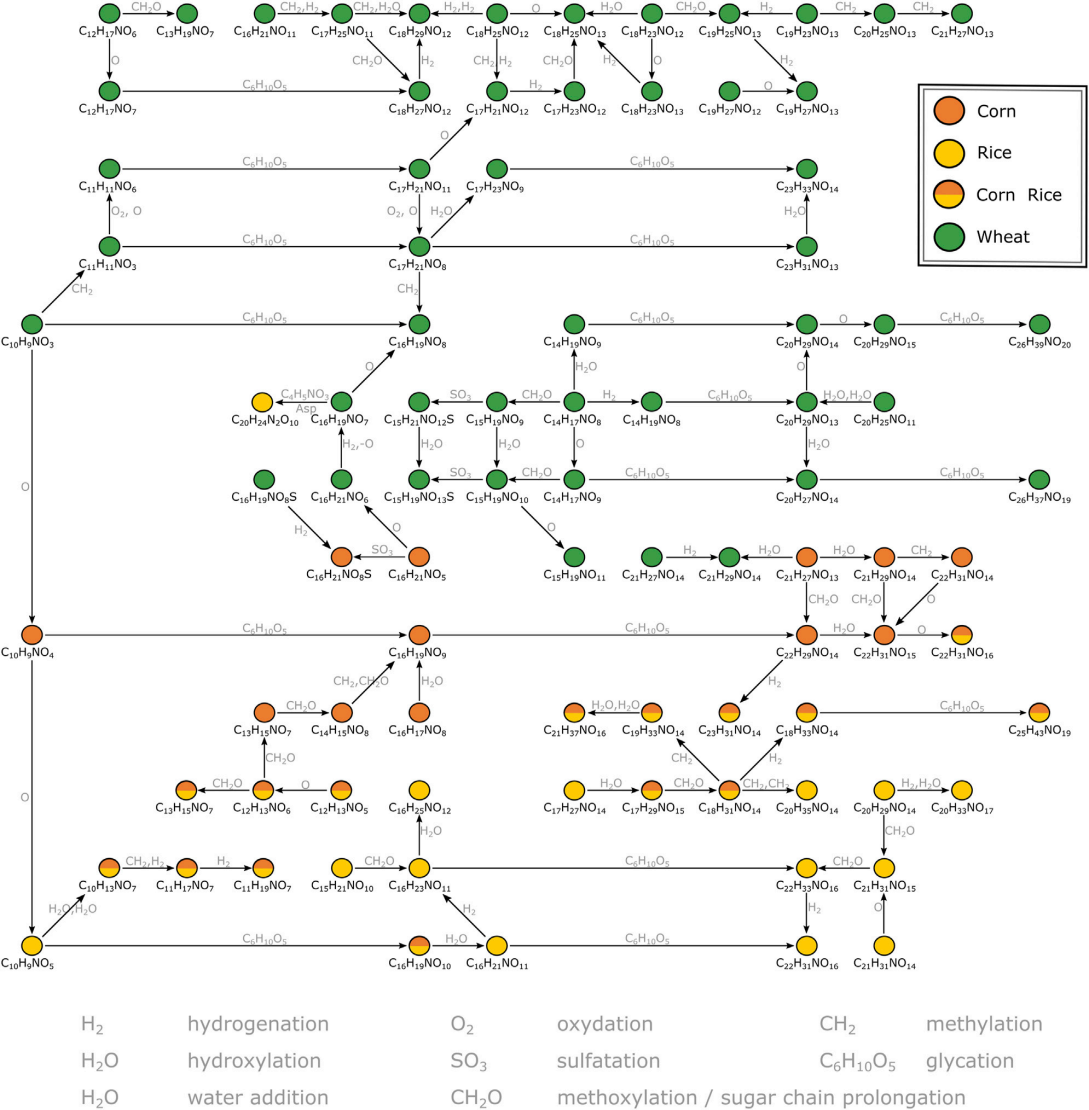
Mass difference network excerpt of compositions characteristic for wheat, corn and rice. The nodes representing annotations are connected by edges representing potential biochemical reactions. Some connections are neglected for reasons of clarity. The annotations likely correspond to secondary metabolites deriving from the indolacetic acid and benzoxazinone biosynthetic pathways respectively.

A second MDiN excerpt that sets wheat, corn and rice markers in biochemical relation was investigated in more detail (Figure 3). As reported before (Pieczonka et al., 2020), the metabolome wheat adds to the beer’s complexity specifically is characterized by compositions corresponding to benzoxazinone derivatives. The mass corresponding to possible blepharin (C_14_H_17_NO_8_), a plant phytoanticipine, is connected to the related HMBOA-glucoside (C_15_H_19_NO_9_) by methoxylation. A subsequent sulfatation gives (C_15_H_21_NO_12_S). An equivalent pattern links the hydroxylated DHBOA-glc (C_14_H_17_NO_9_), the DIMBOA-glc (C_15_H_19_NO_10_) and the respective sulfate (C_15_H_21_NO_13_S). Besides methoxylation, hydroxylation, sulfatation and water addition, several glycation reactions of described molecular formulae lead to a complex network of known and unknown compounds specific for wheat. Those reach from rather unsaturated compositions (e.g., C_10_H_9_NO_3_ and C_11_H_11_NO_3_) to very polar glucosides of the potential aglyca (e.g., C_23_H_31_NO_13_, C_26_H_39_NO_20_ and C_26_H_37_NO_19_). A similar, but smaller, network is being build up for corn based on compounds likely arising from the indoleacetic acid (IAA) biosynthetic pathway. Chloride adduct formation of the respective aglycon hydroxyoxindoleacetic acid (C_10_H_9_NO_4_) reinforces the presence of a carboxylic acid group of these compounds on the molecular structure level. Based on this annotation, known to be specific for the use of corn (Fenz, 1991), two glycation reactions lead to the respective derivatives (C_16_H_19_NO_9_) and (C_22_H_29_NO_14_). Again, several compositional changes equivalent to hydroxylation, hydrogenation, methoxylation or water addition form a network of masses specific to corn. In parallel, there is a similarly structured network starting from (C_10_H_9_NO_5_) for rice. The composition could potentially be annotated to hydroxydioxindoleacetic acid, found in rice bran by Kinashi et al. (1976). The described biochemical relations again lead to compositions specific for rice (e.g., C_16_H_21_NO_11_, C_22_H_31_NO_16_, C_16_H_23_NO_11_, C_22_H_33_NO_16_), but also includes compositions characteristic for corn and rice (e.g., C_10_H_13_NO_7_ and C_16_H_19_NO_10_). Overall, secondary metabolites deriving from tryptophan dependent pathways drive the differentiation of the carbohydrate sources wheat, corn and rice for brewing. The metabolites cover a wide range of polarity, all accessible from direct infusion with FTICR-mass spectrometry. However, metabolites could only be annotated by exact masses and their biochemical relations (expressed as mass differences) in combination with database and literature data. For definite structural confirmation, a chromatography-coupled mass spectrometric approach including ion fragmentation is necessary (UPLC-ToF-MS).

### UPLC-ToF-MS

A representative sub-sample set (100 samples) was treated by solid phase extraction (SPE) and subjected to reversed phase UPLC-ToF-MS. An average of 680 chromatographic features per sample were obtained after applying filter criteria. The peaks shared by at least one third of all beer samples within a carbohydrate source class (1750 peaks) were used for statistical analysis (OPLS-DA). The classification power of the model was highly significant (Supplementary Table 5). The Q^2^ value for quality of prevision (>0.6) and the R^2^Y value for the goodness of the fit (>0.85) prove the statistical relevance whereas overfitting was excluded by CV-ANOVA (*p*-value <<0.05) (Golbraikh and Tropsha, 2002; Westerhuis et al., 2008). As for the FTICR-MS data, the associated score plot (Figure 2B) showed a clear differentiation of barley, wheat, corn and rice beers. In the first two principal components, beers brewed with barley only are separated from wheat beers. Again, beers brewed with corn or rice are agglomerated against the two others (Figure 2B-I).

The third component enables the differentiation of corn and rice beers (Figure 2B-II). Ultimately, a statistical model distinguishing all carbohydrate sources could also be achieved by the isomeric resolved UPLC-ToF-MS data of the pretreated sub-sample set. The metabolite features that drive the separation were extracted from the respective loadings plot accordingly (Supplementary Figure 2).

**FIGURE 4:**
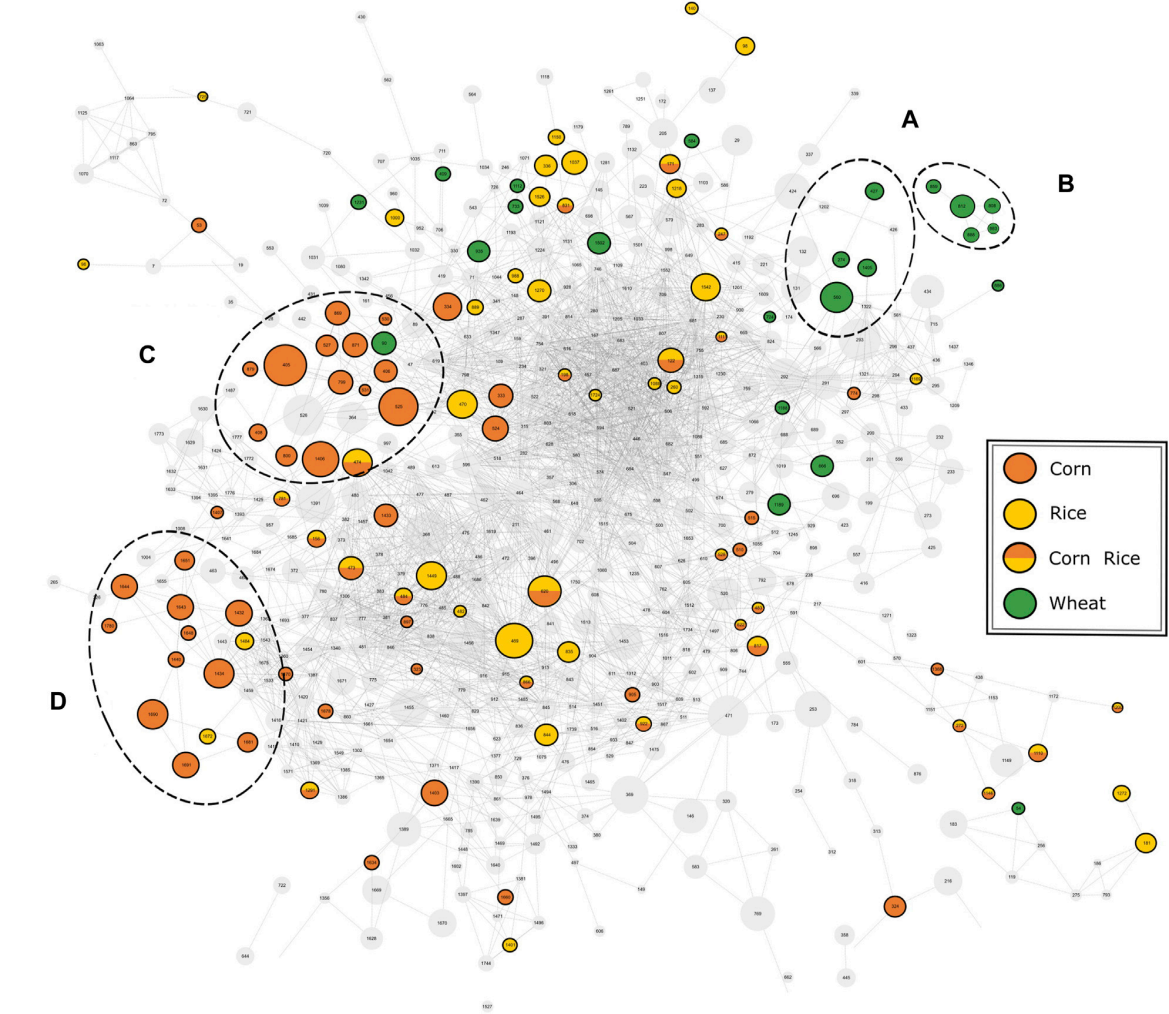
Mass spectral similarity network of the fragmentation spectra of compounds detected by UPLC-ToF-MS. The nodes representing the respective compounds are connected by edges representing their spectral similarity. Compounds found to be specific for a carbohydrate source are colored accordingly. Two cluster of potential marker are highlighted for wheat **(A, B)** and corn **(C, D)**.

The available MS^2^-spectra of the potential marker compounds were utilized in a mass spectral similarity network (Figure 4). The fragmentation spectra of compounds that were examined in more detail can be found in the Supplementary information (Supplementary Table 7). Two clusters with similar fragmentation patterns could be observed for the wheat-specific compounds. The first cluster (Figure 4A) could be identified as an agglomeration of benzoxazinone derivatives, known to be phytoanticipines in the wheat plant and validating the findings with DI-FTICR-MS data. By database and literature research (Bonnington et al., 2003; de Bruijn et al., 2016; Pieczonka et al., 2020) four compounds could be identified as MOBA, HBOA-glucoside, DIBOA-glucoside and HMBOA-glucoside [identification level 2 (Sumner et al., 2007)]. For the second cluster (Figure 4B), only *in silico* fragmentation spectra of database entries were available. All matching spectra found for the five peaks indicate the potential compound class of N-acyl-glutamines (identification level 3). Their almost identical retention behavior (4.32–4.62 min) and proposed molecular formulae [(C_23_H_38_N_2_O_5_), (C_23_H_40_N_2_O_5_), (C_25_H_40_N_2_O_4_), (C_23_H_38_N_2_O_6_) and (C_23_H_40_N_2_O_6_)] support their close chemical relation.

For corn markers, two clusters of compounds with related fragmentation spectra and thus similar structure and origin could be observed. The cluster with the greater significance (Figure 4C) was studied in more detail. The nine compounds examined were found to be isomeric pairs of *m/z*-values matching the molecular formulae (C_20_H_34_O_4_), (C_20_H_32_O_5_), (C_21_H_36_O_4_) and (C_21_H_34_O_5_). The close retention time window between 6.0 and 7.1 min supports their close chemical relation. Together with the similar molecular composition and fragmentation spectra, it brings us to suggest a shared compound class of a non-polar character. The best hits with regard to *in silico* fragmentation spectra all agree on lipid-type structures for the mentioned compounds. Besides, the fragmentation pattern of (C_20_H_34_O_4_) and (C_20_H_32_O_5_) compounds show a great similarity to DiHEtrE and TriHETE fragmentations spectra respectively, when compared to literature data (Wheelan et al., 1996; Ferreiros et al., 2014). However, based on our data, the exact molecular structure and in particular the position of possible hydroxylation cannot be determined. Accordingly, the identification level of this group of corn-specific compounds was indicated to level 3, as suggested by Sumner et al. (2007). In addition to this not yet described cluster of lipid-type molecules, we were able to confirm the hydroxyoxindol-acetic acid as a marker substance for the use of corn by comparison of both *in silico* and literature fragmentation data (Fenz, 1991) (identification level 2).

**FIGURE 5:**
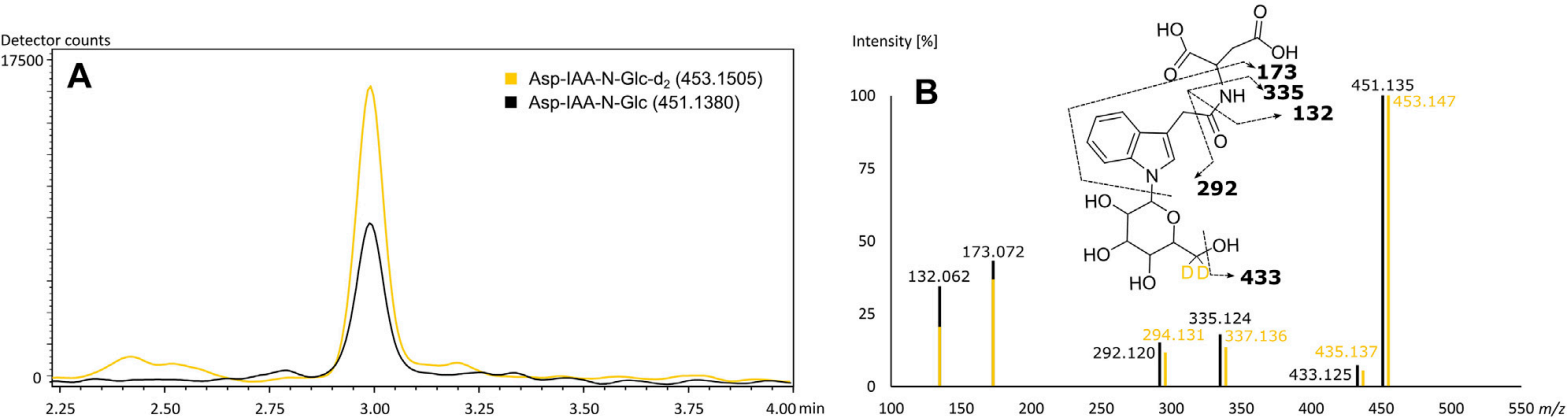
Co-chromatography of the (6, 6-d_2_) N-β-D-glucopyranosyl-indole-3-acetic standard and its isotopologue naturally occurring in beer **(A)** with matching MS^2^-fragmentation spectra in ESI-negative **(B)**. Extracted ion chromatograms of the corresponding m/z values of Asp-IAA-N-Glc-d_2_ (yellow) and of Asp-IAA-N-Glc (black) **(A)**. Mass fragmentation spectra of Asp-IAA-N-Glc-d_2_ (yellow) and of Asp-IAA-N-Glc (black) with corresponding suggested fragments of the mono-isotopologue **(B)**.

The rice specific compounds, few of which were found highly significant in the loadings plot already, did not cluster with regard to their fragmentation pattern. Two of those could be characterized by their molecular formula (C_24_H_40_N_6_O_8_) and (C_22_H_35_N_5_O_11_) as being in accordance with mass values found to be specific in FTICR-MS. A second pair of highly significant peaks could be described as potential Glu-Trp-Leu/Ile-Pro (C_27_H_37_N_5_O_7_) and a cyclic Asp-Ser-Val-Leu-Trp peptide (C_29_H_40_N_6_O_8_), respectively, by comparison of *in silico* fragmentation data (identification level 3). The fifth potential marker of highest interest could be assigned based on both matching fragmentation patterns and co-chromatography (identification level 1). Kai et al. (2007) reported an aspartic acid-conjugate of N-β-D-glucopyranosyl-indole-3-acetic acid to be found in rice with a matching fragmentation pattern. The respective d_2_-standard, synthesized and provided by the mentioned authors, was used for co-chromatography. The rice secondary metabolite was identified by matching retention time and fragmentation pattern (Figure 5). We were able to detect the corresponding peak in the vast majority of rice beers and two beers brewed with corn. To confirm our findings and the origin of the potential marker compounds, we analyzed methanol extracts of food made from the appropriate grain raw materials. Grits, starch and flour of wheat, corn and rice and corn oil were screened for the presence of the specific respective compounds (Supplementary Table 8). Wheat benzoxazinones and potential acyl-glutamines were present in the wheat products. The exception is pure wheat starch, in which none of the compounds were found, as in beers brewed with merely wheat starch (Pieczonka et al., 2020). We were able to confirm the hydroxyoxindol-acetic acid in all corn products except for the oil. The isomeric pairs of lipid class compounds (C_20_H_34_O_4_) and (C_21_H_36_O_4_) were found in the corn oil, whereas the other specific oxygenated lipids might be formed during the brewing process. The same is suspected for the (C_29_H_40_N_6_O_8_) cyclic peptide in rice. The other rice metabolites, including the aspartic acid-conjugate of N-β-D-glucopyranosyl-indole-3-acetic acid, were confirmed by the analysis of rice products. An overlap of the potential markers between the carbohydrate groups was not observed. Interestingly, the coumaryl and caffeoyl glycerols described by Fenz et al. (1992) and confirmed by FTICR-MS were found and identified in the methanol extract of corn grits but not the beers. We assume that they were lost through the SPE sample processing of the beers.

### Comparison and Conclusion

The investigation of the influence of different carbohydrate sources on the metabolome of the beer end product was carried out using two different, complementary mass spectrometric methods. Even with different sample numbers (400 for DI-FTCR-MS and 100 for UPLC-ToF-MS), fundamental differences and commonalities between the analytical approaches could be observed. Based on the direct infusion approach without extensive prior sample preparation, compounds of all polarities (ionizable by ESI) are accessible with FTICR-MS. This is reflected in the MDiN of the secondary metabolites, which maps numerous glycosylation steps up to highly oxygenated compositions. This wide polarity range was not tangible by RP-HPLC-ToF-MS. The corn marker hydroxyoxindol-acetic acid was found with an early retention time (3.02 min), whereas glycosylated derivatives of the aglycone were lost by sample preparation and chromatography. The phosphate-structures found in FTICR-MS could not be found either and thus not be further characterized by fragmentation spectra. The average mass values also differ between FTICR-MS (*m/z* 409) compared to ToF-features (*m/z* 553), which could be attributed to different accessible mass ranges (100 to 1,000 Da for FTICR-MS, 50 to 1,500 Da for ToF-MS). The mass features showed a moderate overlap within a ±5 ppm range, in accordance with the different and complementary chemical spaces analyzed (Supplementary Figure 4). About 35% of the chromatographic features showed had a corresponding *m/z*-value in FTICR-MS, whereas less than 10% of FTICR-MS-masses were found with an equivalent peak in LC-MS. Here, the different numbers of samples should be emphasized again. The majority of the chromatographic peaks showed at least one isomeric compound (up to 16), which confirms the complementarity of the information obtained by coupling chromatography to mass spectrometry. We observed a similarly low *m/z*-overlap with regard to the potential marker features. In particular, it is the statistically most significant compounds, which were detected in large parts in both DI-FTICR-MS and UPLC-ToF-MS. This enabled a deeper characterization through exact mass values and fragmentation mass spectra. Only the group of potential acyl-glutamines and some rice peptide-like structures are not represent in the FTICR-MS-data.

Overall, with two complementary mass spectrometric approaches, we have uncovered deep metabolic signatures that the use of wheat, corn and rice in brewing entails. The majority of the decisive compounds can already be found in the corresponding raw materials and survive the entire brewing process. We were able to set the compositions in relation by mass difference network analysis and uncovered a whole network of secondary metabolites specific to the respective grains. By mass fragmentation, the compounds could be characterized in detail and known reported marker substances could be confirmed. Finally, we want to highlight that in the aspartic acid conjugate of N-β-D-glucopyranosyl-indol-3-acetic acid we report a potential marker for the use of rice in beer.

## Discussion

Both DI-FTICR-MS and UPLC-ToF-MS showed the power of mass spectrometric analysis with regard to food and beverage authenticity. As already shown for wine (Roullier-Gall et al., 2015), the two approaches describe two different and complementary chemical spaces. However, we were able to differentiate simultaneously beers brewed with wheat, corn and rice against those with barley only in both DI-FTICR-MS and UPLC-ToF-MS. The metabolic signatures of the carbohydrate sources commonly used in brewing could be characterized by networks of secondary metabolites, resolved with regard to isomeric distribution and identified by MS^2^-fragmentation information on different levels. Such a comprehensive analysis of grain-specific metabolites was not carried out with regard to barley as all measured beers contained it to various extents and co-varying metadata was observed (Supplementary Figure 1). In addition, beer samples that were brewed with merely wheat starch could not be identified as they lack the grain’s metabolite signature. In the other grain-based foodstuffs, including typical grain adjuncts used in the brewing industry, we were able to detect the potential marker substances to various extents. All compounds could be found at least once, except for two annotated rice (cyclic) peptides and corn lipids, which indicates formation or alteration during the brewing process or insufficiently optimized extraction. Two barley beers of the same non-German brewery were neglected because they showed a clear signature of corn metabolites in spite of the contradicting information in the ingredient list. Although this is an exception, it brings us to the conclusion that, particularly with regard to the use of corn and rice, the metadata of commercial beers must be questioned. An authenticity control of the beer should be considered.

The statistical analysis shows that in both analytical approaches the metabolite profiles of beers brewed with corn and rice are very similar. Only in the third principal component we could tell the respective clusters apart. A possible reason for this could be the closely related genetic evolution (Adams and Wendel, 2005) and botanical relationship (Ahn and Tanksley, 1993) of the plants and thus the similarity of their metabolic signatures even when analyzed in beer. Bearing in mind that barley, wheat, corn and rice all belong to the family of Poaceae and show collinearity (Van Deynze et al., 1995), the reason of the observed similarity may also be found in the similar way of brewing when corn or rice adjuncts are used. Moreover, benzoxazinones found to be specific for wheat beers are not exclusively produced by *Triticum aestivum*, but analogous genetic information is also present in corn (Nomura et al., 2008). This indicates that both the concentrations and the distribution of the secondary metabolites in the plant and thus the parts of the plant that are used for brewing play a decisive role (Koistinen et al., 2020). The more pronounced diversity of benzoxazinone secondary metabolites induced by germination (Mogensen et al., 2006; Koistinen et al., 2020) could also be of importance and a starting point for further authenticity determinations with regard to the use of wheat raw grain adjunct. With this in mind, we hesitate to refer to the grain-specific compounds identified as biomarker molecules. Rather, the aim should be to quantify the metabolites with most sensitive instrumental analytics (e.g., triple quadrupole instruments) in numerous commercial and experimental beers in order to confirm the biomarker nature or define a concentration limit of confidence. Nevertheless, the metabolic signatures found in our study are unambiguous as a whole.

Such compounds that show strong evidence for the use of wheat (Pihlava and Kurtelius, 2016; Pieczonka et al., 2020) and corn (Fenz, 1991; Fenz et al., 1992) in brewing have been adequately described and have been confirmed in our study. In addition, numerous derivatives of these compounds could be characterized. Of particular note are the sulfate derivatives, some of which we already reported (Pieczonka et al., 2020). Little is known about the biological function of these compounds, but their function as polar regulation or storage conjugates can be assumed. Tang et al. (2020) also described these sulfates in human urine samples after wheat intake.

We were able to describe another conjugate of a secondary metabolite as potential marker substance for the use of rice. To the best of our knowledge, literature has not yet reported a rice-specific compound for authenticity control. The aspartic acid conjugate of N-glucosyl-indol-acetic acid was described by Kai et al. (2007) in rice extracts for the first time, as the authors report. In general, IAA is known to regulate many aspects of growth and development in plants. For instance, it is reported to specifically induce a *big grain1* gene, which expresses an auxin transport protein and ultimately results in bigger rice grain size (Liu et al., 2015). In that regard, the concentration of free IAA concentrations is well-balanced by biosynthesis, catabolism and transport mechanisms (Ljung et al., 2001). The conjugation of the auxin to glucose or amino acids is one part of the so-called IAA homeostasis. The aspartic acid and glutamic acid (to a lesser extend) conjugates are found to be the major storage or transport forms of the respective N-glucosyl-indol-acetic acid in rice (Kai et al., 2007). Given that the N-glucoside likely is biosynthesized from the amide conjugates, this relationship could also apply to the reverse. However, the rice characteristic metabolite we found in beer appears to be an inactivated form of the auxin. Kai et al. (2007) described that the free N-glycosyl-indol-3-acetic acid is as well found in corn seedlings and alkaline hydrolysis releases additional amounts (0.45 nmol g^−1^ total amount). However, the conjugated form was not specified and corn grains were not investigated. Accordingly, incorrect information about the ingredients on the beer label of the two potential “corn and barley only” beers cannot be ruled out as all beers were purchased as presented to the consumer. As little is known about the distribution of the auxin derivatives in the plant tissues and thus about the tendency to be extracted during the brewing process, the biomarker character of Asp-IAA-N-Glc needs to be further investigated. With the huge majority of rice beers showing an Asp-IAA-N-Glc signal, a more specific extraction method or more sensitive mass spectrometric approach (e.g., MRM in triple quadrupole) could verify its presence in all beers brewed with rice. We also found the derivative in two corn beers, which might indicate the rare presence of Asp-IAA-N-Glc in corn beers as well. Further investigations must clarify these findings and exclude potential incorrect information on the ingredient list. In any case, the presented potential rice-characteristic compound is, to our knowledge, not found nor reported in either barley or wheat beers or the respective plants.

## Data Availability

The raw data supporting the conclusion of this article will be made available by the authors, without undue reservation.
