# Supplementary material for: On the Trail of the German Purity Law: Distinguishing the Metabolic Signatures of Wheat, Corn and Rice in Beer
Source: Front Chem. 2021 Jul 20;9:715372. doi: 10.3389/fchem.2021.715372 (PMC8329485; doi:10.3389/fchem.2021.715372)
Supplement: Supplementary file 1 [file DataSheet1.pdf]

Supplementary information

On the Trail of the German Purity Law: Distinguishing the Metabolic Signatures of Wheat, Corn and Rice in beer

Stefan A. Pieczonka[1,*], Sophia Paravicini[1], Michael Rychlik[1], Philippe Schmitt-Kopplin[1,2,*]

[1]Chair of Analytical Food Chemistry, Technical University of Munich, Freising, Germany
[2]Research Unit Analytical BioGeoChemistry, Helmholtz Zentrum München, Neuherberg, Germany

Contents

Supplementary tables 2

Supplementary table S1. Carbohydrate source and positions of the beer samples in the multivariate statistical models. 2

Supplementary table S2. SPE work-up of the beer samples for UHPLC-ToF-MS analysis. 5

Supplementary table S3. Parameters for UHPLC-separation and ToF-measurements. 5

Supplementary table S4. Parameters of the UHPLC-ToF-MS data treatment using the MS-Dial software. 6

Supplementary table S5. Statistical parameters of the multivariate data analysis. 6

Supplementary table S6. Mass features resolved by FTICR-MS within the nominal mass m/z 385 throughout the whole sample set. 7

Supplementary table S7. Identification of compounds characteristic for wheat, corn and rice based on UPLC-ToF-MS fragmentation spectra. 9

Supplementary table S8. Presence of the compounds characteristic for wheat, corn and rice in respective grain foodstuff. 10

Supplementary figures 11

Supplementary Figure S1. Score plot of FTICR-MS data (1st and 2nd component) shows the overlap of barley beers (carbohydrate source) with craft beers (beer type). 11

Supplementary Figure 2. Loadings plots of the OPLS-DA of the FI-FTICR-MS **(A)** and UPLC-ToF-MS **(B)** data differentiating the carbohydrate sources used. 12

.... 12

Supplementary Figure S3. Mass difference network excerpt of lipids characteristic for corn. 13

Supplementary Figure S4. Overlap of mass features found in RP-UPLC-ToF-MS (LC-MS) and FI-FITCR-MS (FTICR) within a mass tolerance of 14

Supplementary tables

Supplementary Table S1. Carbohydrate source and positions of the beer samples in the multivariate statistical models.

Sample	Starch source[1]	OPLS scores DI-FTICR-MS components			UHPLC-ToF components			Sample	Starch source[1]	OPLS scores DI-FTICR-MS components			UHPLC-ToF components		
		1st	2nd	3rd	1st	2nd	3rd			1st	2nd	3rd	1st	2nd	3rd
1	W	-10.17	-15.51	-2.89	-	-	-	231	C	-18.73	14.39	-16.69	-	-	-
2	B	12.52	3.04	-3.47	-	-	-	232	R	-11.69	11.14	12.47	-13.66	-0.16	8.98
3	B	12.51	2.05	-3.08	-	-	-	233	B	15.53	4.47	-5.73	7.38	29.16	-2.19
4	B	6.82	3.37	-2.66	-	-	-	234	W	-4.74	-10.08	0.88	27.13	-15.15	-3.49
5	W	-9.66	-21.02	1.51	-	-	-	235	C	-20.27	10.05	-13.60	-	-	-
6	W	-12.96	-19.25	1.08	27.39	-12.75	0.05	236	C	-24.31	12.45	-17.40	-12.69	-5.66	-9.81
7	W	-13.62	-15.55	-1.76	-	-	-	237	C	-22.00	13.69	-19.97	-15.62	-4.96	-14.76
8	B	12.91	4.77	-4.67	-	-	-	238	B	10.52	2.65	-4.97	-	-	-
9	B	13.78	4.32	-4.56	-	-	-	239	R	-12.59	14.95	2.21	-6.62	-5.41	11.35
10	B	13.24	0.79	1.53	-	-	-	241	R	-14.15	15.10	5.54	-13.13	-1.42	8.92
11	W	-11.85	-15.11	-3.19	-	-	-	242	B	10.23	4.35	-3.22	-	-	-
12	W	-6.94	-12.22	0.18	-	-	-	243	B	12.32	1.20	-3.64	-	-	-
13	B	10.69	2.44	-1.17	-	-	-	244	B	9.94	-0.01	-4.07	-	-	-
14	B	4.84	-1.02	-4.54	-	-	-	245	B	6.98	2.75	-1.56	-	-	-
15	B	9.63	0.54	-2.98	-	-	-	246	B	11.09	2.66	-1.81	-	-	-
16	B	13.28	-0.06	-1.19	-	-	-	247	B	4.44	2.31	-2.85	-	-	-
17	C	-	-	-	-8.71	1.66	-14.58	248	B	7.12	1.09	0.79	-	-	-
18	B	9.44	4.01	-1.21	-	-	-	249	B	8.45	2.16	-2.83	-	-	-
19	W	-8.94	-20.70	-1.25	-	-	-	250	W	-10.91	-15.63	-1.58	-	-	-
20	W	-8.29	-13.93	-0.91	-	-	-	251	W	-4.11	-14.67	0.77	-	-	-
21	W	-5.72	-11.01	-0.84	-	-	-	252	B	11.06	2.68	0.88	-0.25	16.35	0.00
22	B	5.59	2.60	-1.54	-	-	-	253	W	-7.29	-21.55	3.42	-	-	-
23	B	10.77	2.02	-1.73	-	-	-	254	B	1.65	3.04	1.81	-	-	-
24	B	6.21	0.44	-0.81	-	-	-	255	W	-8.84	-14.53	0.45	-	-	-
25	W	-8.52	-9.14	-0.51	-	-	-	256	W	-9.50	-11.05	-0.38	23.39	-6.41	-2.31
26	B	11.81	2.94	0.07	-	-	-	257	W	-7.11	-18.75	1.59	26.72	-12.49	-2.96
27	B	11.68	1.50	-0.93	-	-	-	258	W	-6.13	-16.23	3.18	23.75	-5.50	-3.17
28	W	-3.74	-10.63	-0.47	-	-	-	259	W	-5.89	-15.65	1.40	-	-	-
29	W	-6.59	-16.52	2.29	-	-	-	260	B	6.65	-0.04	2.13	-	-	-
30	B	7.55	0.96	-0.91	-	-	-	261	B	8.70	1.69	-0.05	-	-	-
31	W	-5.18	-12.49	-0.94	-	-	-	262	B	8.90	3.73	-0.92	-	-	-
32	B	1.61	2.12	-2.80	-	-	-	263	W	-14.09	-19.62	-0.87	-	-	-
33	W	-8.42	-18.95	1.37	29.37	-8.02	0.55	264	C	-18.04	16.07	-13.07	-12.75	-7.06	-10.38
34	W	-8.56	-15.34	0.73	-	-	-	265	C	-18.25	15.63	-7.33	-	-	-
35	B	11.10	1.40	-1.66	-	-	-	266	C	-19.50	10.38	-7.71	-7.04	-1.53	-8.52
36	B	11.28	-0.62	0.93	3.78	18.89	4.94	267	C	-22.09	13.46	-8.36	-	-	-
37	B	11.82	0.07	-3.80	-	-	-	268	R	-3.17	10.93	9.68	-13.01	2.67	6.66
38	W	-4.43	-14.77	0.92	-	-	-	269	C	-15.07	7.01	-7.50	-2.73	-2.25	-5.99
39	B	8.18	0.82	-2.30	0.72	22.17	-1.55	270	C	-18.38	11.25	-11.27	-10.04	-6.96	-13.37
40	B	15.51	5.52	-2.47	-	-	-	271	C	-13.62	8.49	-9.38	-7.67	-1.82	-5.49
41	W	-8.66	-15.30	-1.60	-	-	-	272	R	-13.05	15.52	6.69	-	-	-
43	B	1.12	0.01	-1.47	-	-	-	273	B	9.04	1.41	-0.96	-	-	-
44	B	10.74	-0.49	-0.77	-	-	-	274	R	-14.59	19.35	13.50	-6.33	-3.95	4.08
45	W	-5.70	-16.65	0.41	-	-	-	275	C	-19.59	17.30	-4.08	-10.47	2.57	-12.69
46	B	4.31	-2.46	-1.22	-	-	-	276	C	-17.75	15.12	-15.38	-8.17	-3.43	-3.16
47	W	-1.26	-9.82	-1.79	-	-	-	277	C	-13.69	13.55	-9.19	-	-	-
48	B	5.37	-0.88	0.19	-	-	-	278	B	4.31	2.80	2.08	-	-	-
49	B	6.75	-0.21	0.41	-	-	-	279	B	2.03	5.58	-1.27	-	-	-
50	B	11.93	4.27	-0.35	-	-	-	280	C	-16.13	12.93	-8.09	-	-	-
51	W	-11.96	-15.43	-0.48	32.21	-3.54	-0.86	281	B	8.57	1.49	0.65	-	-	-
52	W	-10.10	-17.32	1.61	23.18	-16.85	1.09	282	B	11.96	3.77	-1.13	-	-	-
53	B	10.06	2.68	-4.19	-	-	-	283	C	-8.18	11.86	-6.99	-	-	-
54	B	1.92	-2.42	-0.51	-	-	-	284	B	6.75	2.50	-0.07	-	-	-
55	W	-0.89	-12.12	0.45	-	-	-	285	C	-13.94	12.29	-10.30	-12.19	-1.67	-13.21
56	W	-7.74	-17.72	-2.61	-	-	-	286	B	8.14	2.27	2.19	-	-	-
57	B	8.26	0.26	0.81	-	-	-	287	B	6.20	0.83	0.18	-	-	-
58	W	-6.63	-16.50	0.35	-	-	-	288	B	2.87	0.95	-0.59	7.48	21.97	-2.11
59	B	3.16	0.01	-1.76	-	-	-	289	W	-13.96	-8.64	-2.65	-	-	-
60	B	11.52	1.58	-1.34	-	-	-	290	W	-7.47	-12.76	4.01	-	-	-
61	W	-3.40	-13.26	-2.84	-	-	-	291	B	4.15	1.00	1.80	-	-	-
62	W	-9.37	-18.90	-2.35	-	-	-	292	B	10.22	2.35	1.84	-	-	-
63	W	-8.04	-21.04	3.15	-	-	-	293	B	6.47	3.31	-1.90	2.28	19.01	0.35
64	B	11.89	-1.60	1.09	-	-	-	294	B	6.19	2.17	1.11	8.50	20.45	-3.82
65	B	9.29	-0.12	0.73	-	-	-	295	B	4.80	1.29	0.87	-	-	-
66	B	9.30	2.13	-0.63	-	-	-	296	C	-16.08	9.57	-6.27	-11.31	-6.81	-8.04
67	W	-6.47	-15.98	3.09	-	-	-	297	R	-	-	-	-11.43	-1.23	11.16
68	B	5.74	0.03	-0.18	-	-	-	298	B	2.58	1.79	3.13	-	-	-
69	W	-3.11	-11.65	0.29	12.73	-2.80	-1.55	299	W	-3.28	-9.13	1.20	-	-	-
70	B	13.47	-0.21	0.69	-	-	-	300	B	9.72	2.18	-0.03	-	-	-
71	B	7.36	3.29	1.51	5.70	26.70	1.50	301	B	13.75	2.65	3.39	-	-	-
72	B	7.49	3.97	-1.99	-	-	-	302	B	2.10	5.39	-0.98	-	-	-
73	B	6.77	-0.09	0.13	-	-	-	303	W	-7.36	-17.79	2.39	-	-	-
76	W	-6.77	-17.28	0.80	-	-	-	304	B	15.52	3.11	-0.48	-	-	-
78	B	8.18	1.00	3.24	-	-	-	305	W	-10.36	-18.80	2.24	-	-	-
80	R	-6.24	10.88	6.59	-	-	-	306	B	9.92	4.81	3.83	-	-	-
81	B	3.03	-1.42	0.85	-	-	-	307	R	-6.02	14.42	7.21	-7.62	-3.97	7.69
83	B	7.43	0.08	0.52	-	-	-	308	W	-16.52	-19.82	7.12	-	-	-
85	W	-2.96	-8.99	-0.42	-	-	-	309	R	-16.87	7.67	18.03	-12.54	-5.16	6.85
86	B	11.70	-0.15	0.00	11.72	27.44	-0.79	310	B	8.56	1.59	2.23	-	-	-
90	B	11.99	0.92	-5.30	-	-	-	311	R	-3.01	9.01	2.04	-9.40	-1.09	4.71
91	B	11.55	2.63	-5.10	-	-	-	312	R	-1.14	8.32	3.64	-7.04	0.99	5.75
92	B	10.77	1.89	-0.03	-	-	-	313	R	-5.85	13.48	2.55	-14.13	-0.69	6.99
93	B	6.00	3.35	-2.48	-	-	-	314	W	-11.41	-22.25	-0.10	-	-	-
94	B	3.62	0.19	-1.35	-	-	-	315	B	11.94	1.34	-2.65	-	-	-
95	?[2]	14.62	11.13	-2.73	-8.45	-5.69	-12.26	317	R	-14.49	15.44	5.75	-12.39	-5.66	7.79

96	B	9.06	0.08	-2.87	-	-	-	318	R	-14.19	19.57	19.00	-	-	-
97	C	-24.39	13.00	-14.70	-9.50	-1.96	0.40	319	C	-24.18	17.38	-18.15	-13.85	-4.52	-8.84
98	B	6.07	1.04	-2.13	-	-	-	320	C	-22.83	15.17	-9.88	-11.34	-10.16	-4.05
99	B	13.66	8.35	-0.52	-	-	-	321	R	-7.26	13.50	6.68	-4.63	1.92	8.93
101	R	-18.30	18.25	26.14	-	-	-	322	R	-12.18	18.63	15.72	-9.45	-3.64	6.64
102	W	-13.43	-19.55	1.58	-	-	-	323	C	-19.29	13.68	-15.29	-	-	-
103	B	10.37	-1.35	-2.45	-	-	-	324	R	-19.93	21.34	13.25	-12.77	-1.50	8.42
104	B	11.51	2.16	-6.88	-	-	-	325	R	-4.86	10.68	19.95	-9.04	-1.23	14.22
105	W	-7.88	-17.88	-0.44	-	-	-	326	B	4.65	1.47	-3.18	-	-	-
106	W	-7.41	-16.59	-1.48	-	-	-	327	R	-16.93	19.47	9.50	-	-	-
107	B	8.99	1.26	-2.46	-	-	-	328	C	-24.98	13.26	-10.93	-	-	-
108	W	-9.76	-18.63	1.97	-	-	-	329	C	-20.38	14.34	-12.91	-14.79	-6.03	-8.37
109	B	7.39	2.91	-2.80	-	-	-	330	C	-25.39	16.05	-14.58	-	-	-
110	B	9.67	-2.63	0.83	-	-	-	331	R	-10.67	16.73	17.68	-13.69	-7.56	14.09
111	B	12.12	-0.35	-4.84	-	-	-	333	B	2.03	7.34	-1.75	-	-	-
112	B	6.44	0.04	3.26	-	-	-	334	R	-14.52	18.71	21.59	-5.18	-2.40	10.09
113	B	9.44	-2.37	1.11	6.51	30.10	1.62	335	R	-19.21	19.18	24.29	-	-	-
114	B	3.20	3.05	-1.83	-	-	-	336	R	-12.99	14.46	19.36	-3.84	-5.22	13.00
115	W	-10.76	-11.56	-0.27	13.73	-12.35	-1.54	337	R	-15.92	19.22	23.25	-14.85	-8.10	4.42
116	B	8.62	-0.29	-1.57	-	-	-	345	C	-11.27	15.93	-9.72	-10.17	-3.06	-8.05
117	W	-9.03	-18.22	1.82	-	-	-	351	W	-4.01	-11.76	-1.26	18.45	-10.12	2.70
118	W	-8.42	-15.82	1.77	22.76	-6.06	5.83	352	W	-4.89	-19.97	2.63	24.34	-7.36	1.25
119	B	5.09	-0.66	-0.43	-	-	-	354	W	1.01	-1.06	-5.06	20.63	-11.14	0.94
121	W	-7.53	-11.73	-1.18	-	-	-	357	W	-13.40	-19.18	3.30	-	-	-
123	W	-3.10	-8.23	-0.23	-	-	-	358	B	8.75	3.20	-0.77	0.37	20.15	2.91
124	W	-7.25	-13.71	-0.73	-	-	-	360	C	-	-	-	-7.91	-9.04	-4.65
125	B	13.47	1.49	2.76	-	-	-	364	W	-9.00	-8.69	6.60	-	-	-
126	W	-7.98	-16.55	-0.03	-	-	-	365	B	10.60	1.37	0.56	-	-	-
127	B	11.73	2.54	-2.62	-	-	-	366	R	-13.41	20.07	11.36	-15.25	-5.07	8.65
128	B	11.24	-2.33	0.81	-	-	-	367	W	-11.53	-17.33	5.13	17.05	-10.23	-0.96
129	B	9.33	-3.86	-1.26	-	-	-	368	B	9.30	6.87	-6.00	-	-	-
130	B	9.75	1.07	-0.19	-	-	-	369	B	9.96	3.89	-0.58	-	-	-
131	B	4.16	-1.38	2.16	-	-	-	370	B	13.14	-0.16	0.87	-	-	-
132	B	10.36	0.48	1.06	-	-	-	371	W	-7.97	-18.90	3.10	28.88	-14.24	-0.87
133	W	-1.99	-15.74	4.15	-	-	-	372	W	-14.11	-19.18	4.57	-	-	-
134	B	12.90	4.48	-4.32	-	-	-	375	B	12.13	4.14	-1.52	-	-	-
136	B	5.58	2.42	-5.36	-	-	-	376	B	8.95	0.28	-7.38	-	-	-
137	W	-5.15	-17.09	-3.08	-	-	-	379	C	-24.06	15.98	-21.73	-3.64	0.86	-2.36
138	W	-4.78	-16.36	3.84	-	-	-	380	B	14.42	4.03	-0.11	-	-	-
139	B	13.74	-3.22	-0.44	-	-	-	381	R	-11.35	14.83	13.09	-	-	-
140	B	14.51	1.68	-2.39	-	-	-	382	R	-9.97	13.37	15.02	-3.77	-8.10	6.01
141	B	11.58	0.72	-5.31	-	-	-	383	C	-21.36	16.40	-10.11	-	-	-
142	B	10.11	2.96	1.34	-	-	-	385	B	17.54	-2.41	-7.06	-	-	-
143	B	15.90	0.31	-1.23	-	-	-	387	B	13.67	2.95	-2.59	-	-	-
144	B	10.23	3.85	-0.46	-	-	-	389	B	10.63	3.91	0.88	-	-	-
145	B	4.53	2.71	1.64	-	-	-	390	B	9.44	4.00	1.33	-	-	-
146	B	6.76	1.44	-0.38	10.08	26.56	-6.01	391	R	-15.60	16.05	13.59	-	-	-
147	B	10.92	0.71	-2.59	-	-	-	392	B	12.10	3.02	3.06	-	-	-
148	B	9.12	0.00	-1.85	8.01	26.50	2.20	393	B	6.72	0.34	0.63	-	-	-
149	?[2]	10.16	-1.77	-14.73	5.07	7.07	-14.59	394	B	-	-	-	6.79	19.17	-0.90
151	C	-13.99	7.89	-11.48	-10.57	-4.83	-11.33	395	W	-12.37	-17.87	-0.90	-	-	-
152	W	-7.77	-18.24	4.39	16.65	-9.37	0.81	396	B	9.02	3.26	2.59	-	-	-
153	B	4.09	2.67	-2.73	-	-	-	397	W	-7.40	-16.13	2.27	-	-	-
154	B	7.68	5.30	-2.03	-	-	-	398	B	9.61	1.26	0.30	-	-	-
155	W	-13.21	-21.20	3.11	28.79	-13.35	3.49	399	B	10.69	-2.68	2.02	-	-	-
156	C	-13.20	6.74	-11.84	-	-	-	400	W	-9.85	-20.28	5.49	-	-	-
157	B	9.92	3.12	-1.17	-	-	-	401	B	7.38	-2.79	2.23	-	-	-
159	B	16.03	0.63	-5.86	-	-	-	402	W	-9.82	-15.69	2.45	-	-	-
160	C	-22.79	12.01	-18.87	-	-	-	404	W	-1.33	-7.90	0.53	-	-	-
162	B	12.62	0.13	2.43	-	-	-	405	W	-9.34	-13.19	3.55	-	-	-
163	B	10.66	1.75	-0.25	-	-	-	406	B	8.65	-1.72	2.57	-	-	-
164	C	-23.51	11.99	-13.87	-6.80	-7.15	-5.68	407	R	-13.66	14.27	13.77	-	-	-
165	B	5.99	5.31	0.68	-	-	-	408	C	-	-	-	-12.23	-1.76	-1.37
166	?[2]	-9.18	15.07	2.27	-14.96	-1.74	1.23	409	W	-13.37	-23.69	-0.99	-	-	-
167	C	-16.64	13.28	-4.16	-8.34	3.76	1.24	410	R	-13.06	17.89	20.86	-11.96	-4.11	10.12
168	R	-10.77	12.65	13.34	-	-	-	411	C	-25.87	13.57	-18.47	-8.17	-5.90	-5.60
169	C	-13.20	12.15	-8.45	-	-	-	412	W	-12.11	-16.23	4.34	-	-	-
170	R	-3.89	11.07	6.34	-	-	-	413	R	-12.62	16.33	17.63	-	-	-
171	R	-7.03	14.15	7.27	-	-	-	414	B	11.63	2.17	-1.84	-	-	-
172	C	-14.62	13.55	-8.73	-	-	-	416	B	9.41	-1.47	-0.23	-	-	-
173	R	-11.25	13.81	5.86	-9.02	-0.90	8.04	417	B	7.80	1.33	1.51	-	-	-
174	B	9.87	0.80	3.86	10.40	26.11	2.35	418	B	12.42	4.72	0.82	-	-	-
175	B	4.61	1.46	0.81	9.14	17.90	9.28	419	B	8.57	4.09	1.11	-	-	-
177	B	10.19	-1.01	2.80	-	-	-	420	B	10.35	2.25	-2.12	-	-	-
178	B	7.37	4.22	-0.11	-	-	-	421	B	7.86	-0.69	-2.28	-	-	-
179	B	3.79	3.96	0.57	-	-	-	422	B	6.91	1.13	1.47	-	-	-
180	B	6.84	0.26	-4.08	-	-	-	424	B	6.87	1.49	4.28	-	-	-
182	B	9.41	3.02	4.40	-	-	-	425	B	6.28	1.54	1.85	-	-	-
191	C	-13.70	11.39	-12.70	-12.92	-1.73	-8.99	426	B	19.33	-0.64	-3.71	-	-	-
192	B	13.19	2.03	3.89	-	-	-	427	B	9.24	3.69	0.75	11.34	19.01	-1.06
193	B	10.37	-0.50	-3.57	-	-	-	428	B	8.73	0.75	1.76	-	-	-
194	W	-10.53	-15.45	-4.21	-	-	-	429	B	5.78	5.34	-0.68	-	-	-
197	B	4.59	0.53	-0.75	-	-	-	430	B	9.81	1.43	0.33	-	-	-
198	W	-6.23	-8.59	-0.74	14.31	-8.58	-5.95	431	B	4.95	1.52	-0.15	-	-	-
199	B	12.61	1.55	2.35	-	-	-	432	B	9.38	1.65	-0.95	-	-	-
200	W	-15.96	-18.50	0.21	24.60	-17.21	0.77	433	B	16.87	6.85	-5.87	-	-	-
201	B	8.63	5.08	-1.72	-	-	-	434	B	14.37	3.77	3.53	-	-	-
202	W	-12.53	-20.72	4.45	19.55	-7.58	-0.22	435	B	10.42	-1.48	1.29	8.84	22.96	-1.22
203	B	10.40	3.99	1.49	-	-	-	436	B	10.74	-0.94	1.62	-	-	-
204	C	-18.32	6.75	-9.53	-13.68	-0.04	-1.84	437	R	-12.37	17.13	18.95	-	-	-
205	B	4.91	5.83	2.46	-	-	-	438	B	12.49	4.63	-3.96	-	-	-
206	B	5.92	0.71	-2.09	-	-	-	439	W	-1.64	-11.50	0.67	-	-	-
207	W	-6.77	-17.18	4.71	-	-	-	440	B	7.11	2.96	-4.13	-	-	-
208	B	10.39	4.06	0.52	-	-	-	441	W	-14.15	-21.57	2.26	-	-	-
209	B	5.74	2.54	2.11	-	-	-	442	W	-11.55	-14.12	-0.68	-	-	-
210	B	13.12	7.44	-1.10	-	-	-	443	B	8.27	2.67	-2.84	-	-	-
211	B	13.72	3.06	1.74	-	-	-	444	B	5.46	-1.93	-1.57	-	-	-
212	B	4.96	-2.26	1.92	-	-	-	445	B	13.72	4.17	-5.14	4.37	21.39	-1.79
213	R	-12.88	19.82	17.69	-14.38	-4.99	11.49	446	W	-9.56	-13.93	-1.58	-	-	-
214	B	6.87	2.54	-3.27	-	-	-	447	B	9.14	1.58	1.14	-	-	-

215	B	12.25	1.89	0.52	-	-	-	448	B	7.70	0.46	-0.44	-	-	-
216	B	9.76	2.12	-2.14	-	-	-	449	W	-6.07	-13.26	3.26	10.42	-8.71	-0.01
217	W	-12.08	-19.90	2.43	27.19	-12.02	1.09	450	W	-8.42	-15.81	3.65	16.48	-0.98	-1.69
218	B	11.81	1.46	-0.90	-	-	-	451	B	7.74	5.49	-3.52	12.86	25.64	-3.92
219	B	11.69	1.35	-0.79	-	-	-	452	R	-8.67	9.82	13.53	-	-	-
220	B	11.00	-0.35	-3.86	-	-	-	453	W	-6.69	-16.87	5.69	-	-	-
221	W	-7.45	-13.45	-1.66	-	-	-	454	B	6.08	3.42	2.69	-	-	-
222	R	-5.91	15.26	11.57	-14.04	-7.45	10.75	455	B	10.54	7.25	2.85	-	-	-
223	B	10.33	3.44	-4.12	-	-	-	456	B	5.42	2.35	0.10	-	-	-
224	B	10.64	3.28	1.18	-	-	-	457	B	13.21	1.41	2.34	-	-	-
226	B	8.56	1.12	-4.46	-	-	-	458	B	7.66	0.81	2.74	-	-	-
227	W	-13.81	-20.03	1.48	-	-	-	459	B	9.85	6.32	2.27	-	-	-
228	C	-19.75	12.60	-15.16	-16.13	-6.11	-12.60	464	R	-8.90	14.38	14.69	-	-	-
229	C	-18.34	11.72	-12.58	-12.26	-1.58	-13.79	465	R	-5.39	13.22	9.88	-	-	-
230	C	-8.36	9.60	-9.01	-17.72	-8.35	-9.21	467	R	-5.40	14.15	13.61	-	-	-

[1] barley (B), wheat (W), corn (C) and Rice (R)

[2] The sample was excluded from the models because of contradicting information on the beer bottle and unambiguous positions in the score plots (FTICR and UPLC-MS). Only predicted score values are available for the sample.

Supplementary Table S2. SPE work-up of the beer samples for UHPLC-ToF-MS analysis.

Cartridge	Bond Elut PPL, 1 mL and 100 mg (Agilent Santa Clara, CA, USA)
conditioning	1000 µL MeOH<br>2x 1000 µL Milli-Q Water + 2 % FA
sample	1000 µL acidified sample (2 % FA)
washing	500 µL Milli-Q Water + 2 % FA
dry vacuum	
elution	2x 500 µL MeOH

Supplementary table S3. Parameters for UHPLC-separation and ToF-measurements.

Parameter	Value
sample preparation	Supplementary table S2.
column	RP (C18: 1.7 µm, 2.1 x 100 mm, Acquity™ UPLC BEH™)
flow rate	400 µL min^{-1}
column temperature	40 °C
injection volume	5 µL (partial loop)
gradient profile	95 % A (0.1 % formic acid in water) and 5 % B (0.1 % formic acid in acetonitrile) for 1 min; decreasing to 0.5 % A in 5 min; held for 4 min.
measurement time	10 min.
internal calibration	ESI-L Low Concentration Tuning Mix
external calibration	Sodium iodide solution clusters
ESI ionization mode	negative
nitrogen flowrate	10 L min^{-1}
interface temperature	300°C
nebulizer gas flow	1 L min^{-1}
interface voltage	-4 kV
DL temperature	250 °C
Heat block temperature	400 °C
drying gas flow	10 L min^{-1}
detector voltage	2 kV
MS^1 parameters	5 Hz event cycle time
	Ion accumulation on
MS^2 fragmentation parameters	DDA (3 dependent events)
	CE spread 20 eV ± 15 eV

Supplementary Table S4. Parameters of the UHPLC-ToF-MS and FTICR-MS data treatment using the MS-Dial and SIMCA software.

Parameter (LC-MS-data treatment)	value
Minimum peak height	1800 ampl.
Minimum peak width	10 scans
RT tolerance	3 sec.
m/z tolerance	0.005 Da
Sample Intensity / blank Intensity	>10 fold change
Filter	>33 % of the samples within the given class
Normalization (compensation for intensity fluctuations)	LOWESS, based on 22 QCs
MS/MS network similarity	65% cutoff
Zero filling	Random value between average minimum peak intensity for each sample ± σ, (LC-MS and FT-MS)
Normalization	Z-scores (LC-MS and FT-MS)
Scaling	Unit-Variance (UV) scaling (LC-MS and FT-MS)
Transformation	Logarithmize (only applied to FT-MS)

Supplementary Table S5. Statistical parameters of the multivariate data analysis.

Statistical model	n	features	Components (predictive + orthogonal)	R2Y	R2X	Q^2	ANOVA
OPLS (FTICR-MS)	392	7697	3+9	0.881	0.378	0.601	<< 0.05
OPLS (UHPLC-ToF-MS)	98	1776	3+5	0.862	0.528	0.641	<< 0.05

Supplementary Table S6. Mass features resolved by FTICR-MS within the nominal mass m/z 385 throughout the whole sample set.

m/z	neutral Formula	mean intensity	loadings		marker
			3rd component	2nd component	
385.02360	$C_{15}H_{14}O_{10}S$	5.8E+06	-0.054	0.020	Corn
385.03811	$C_{11}H_{18}N_2O_9S_2$	3.9E+06	-0.025	0.006	Corn
385.04461	$C_{12}H_{18}O_{12}S$	6.9E+06	-0.005	0.001	
385.04604	$C_{13}H_{14}N_4O_8S$	5.8E+06	0.003	0.000	
385.04932	$C_{10}H_{18}N_4O_8S_2$	4.8E+06	-0.021	0.002	Corn
385.05412	$C_{12}H_{19}O_{12}P$	1.2E+07	-0.001	-0.010	
385.07459	$C_{12}H_{22}N_2O_8S_2$	1.1E+07	-0.006	-0.002	
385.07764	$C_{16}H_{18}O_{11}$	8.3E+06	0.015	-0.017	Wheat
385.08091	$C_{13}H_{22}O_{11}S$	4.6E+06	-0.020	0.001	Corn
385.08888	$C_{15}H_{18}N_2O_{10}$	8.3E+06	0.007	0.020	Corn&Rice
385.09291	$C_{20}H_{18}O_8$	6.8E+06	0.013	0.001	
385.09876	$C_{13}H_{22}O_{13}$	8.2E+06	-0.001	-0.005	
385.10023	$C_{14}H_{18}N_4O_9$	7.9E+06	0.002	0.000	
385.10179	$C_{12}H_{23}N_2O_{10}P$	1.0E+07	-0.007	-0.007	
385.11404	$C_{17}H_{22}O_{10}$	6.6E+07	0.001	-0.018	Wheat
385.11547	$C_{18}H_{18}N_4O_6$	2.2E+07	0.048	0.009	Rice
385.11733	$C_{14}H_{26}O_{10}S$	6.9E+06	-0.007	0.006	
385.12525	$C_{16}H_{22}N_2O_9$	6.9E+06	-0.002	-0.010	
385.13259	$C_{18}H_{26}O_7S$	1.6E+07	0.004	0.021	Corn&Rice
385.13496	$C_{14}H_{26}O_{12}$	1.3E+07	0.000	0.003	
385.13649	$C_{15}H_{22}N_4O_8$	4.1E+07	-0.001	-0.008	
385.13816	$C_{13}H_{27}N_2O_9P$	6.2E+07	0.027	0.015	Rice
385.14634	$C_{13}H_{26}N_2O_{11}$	9.3E+06	0.008	-0.018	Wheat
385.14941	$C_{12}H_{27}N_4O_8P$	6.8E+06	0.003	-0.016	Wheat
385.15042	$C_{18}H_{26}O_9$	6.4E+06	-0.003	-0.006	
385.16161	$C_{17}H_{26}N_2O_8$	8.7E+06	-0.001	-0.010	
385.16563	$C_{22}H_{26}O_6$	6.6E+06	0.013	0.009	
385.17140	$C_{15}H_{30}O_{11}$	1.3E+07	-0.009	-0.022	Wheat
385.17288	$C_{16}H_{26}N_4O_7$	2.3E+07	0.008	0.024	Corn&Rice
385.17867	$C_{20}H_{30}O_5Cl$	6.6E+06	-0.005	0.008	
385.18676	$C_{19}H_{30}O_8$	6.8E+06	0.006	-0.001	
385.19801	$C_{18}H_{30}N_2O_7$	5.1E+06	0.029	0.003	Rice
385.20201	$C_{23}H_{30}O_5$	4.3E+06	0.001	0.001	
385.20923	$C_{17}H_{30}N_4O_6$	9.1E+06	0.005	0.032	Corn&Rice
385.22314	$C_{20}H_{34}O_7$	2.3E+07	-0.007	0.005	
385.23840	$C_{24}H_{34}O_4$	9.2E+06	0.005	-0.010	
385.24560	$C_{18}H_{34}N_4O_5$	4.8E+06	-0.011	0.009	Corn
385.25953	$C_{21}H_{38}O_6$	7.5E+06	0.021	0.009	Rice
385.29592	$C_{22}H_{42}O_5$	8.0E+06	0.015	0.001	
385.33232	$C_{23}H_{46}O_4$	6.4E+06	0.009	0.006	
385.02360	$C_{15}H_{14}O_{10}S$	5.8E+06	-0.054	0.020	Corn
385.03811	$C_{11}H_{18}N_2O_9S_2$	3.9E+06	-0.025	0.006	Corn
385.04461	$C_{12}H_{18}O_{12}S$	6.9E+06	-0.005	0.001	
385.04604	$C_{13}H_{14}N_4O_8S$	5.8E+06	0.003	0.000	
385.04932	$C_{10}H_{18}N_4O_8S_2$	4.8E+06	-0.021	0.002	Corn
385.05412	$C_{12}H_{19}O_{12}P$	1.2E+07	-0.001	-0.010	
385.07459	$C_{12}H_{22}N_2O_8S_2$	1.1E+07	-0.006	-0.002	
385.07764	$C_{16}H_{18}O_{11}$	8.3E+06	0.015	-0.017	Wheat
385.08091	$C_{13}H_{22}O_{11}S$	4.6E+06	-0.020	0.001	Corn
385.08888	$C_{15}H_{18}N_2O_{10}$	8.3E+06	0.007	0.020	Corn&Rice
385.09291	$C_{20}H_{18}O_8$	6.8E+06	0.013	0.001	
385.09876	$C_{13}H_{22}O_{13}$	8.2E+06	-0.001	-0.005	
385.10023	$C_{14}H_{18}N_4O_9$	7.9E+06	0.002	0.000	
385.10179	$C_{12}H_{23}N_2O_{10}P$	1.0E+07	-0.007	-0.007	
385.11404	$C_{17}H_{22}O_{10}$	6.6E+07	0.001	-0.018	Wheat
385.11547	$C_{18}H_{18}N_4O_6$	2.2E+07	0.048	0.009	Rice
385.11733	$C_{14}H_{26}O_{10}S$	6.9E+06	-0.007	0.006	
385.12525	$C_{16}H_{22}N_2O_9$	6.9E+06	-0.002	-0.010	
385.13259	$C_{18}H_{26}O_7S$	1.6E+07	0.004	0.021	Corn&Rice
385.13496	$C_{14}H_{26}O_{12}$	1.3E+07	0.000	0.003	
385.13649	$C_{15}H_{22}N_4O_8$	4.1E+07	-0.001	-0.008	
385.13816	$C_{13}H_{27}N_2O_9P$	6.2E+07	0.027	0.015	Rice
385.14634	$C_{13}H_{26}N_2O_{11}$	9.3E+06	0.008	-0.018	Wheat
385.14941	$C_{12}H_{27}N_4O_8P$	6.8E+06	0.003	-0.016	Wheat
385.15042	$C_{18}H_{26}O_9$	6.4E+06	-0.003	-0.006	
385.16161	$C_{17}H_{26}N_2O_8$	8.7E+06	-0.001	-0.010	
385.16563	$C_{22}H_{26}O_6$	6.6E+06	0.013	0.009	
385.17140	$C_{15}H_{30}O_{11}$	1.3E+07	-0.009	-0.022	Wheat
385.17288	$C_{16}H_{26}N_4O_7$	2.3E+07	0.008	0.024	Corn&Rice
385.17867	$C_{20}H_{30}O_5Cl$	6.6E+06	-0.005	0.008	
385.18676	$C_{19}H_{30}O_8$	6.8E+06	0.006	-0.001	
385.19801	$C_{18}H_{30}N_2O_7$	5.1E+06	0.029	0.003	Rice
385.20201	$C_{23}H_{30}O_5$	4.3E+06	0.001	0.001	
385.20923	$C_{17}H_{30}N_4O_6$	9.1E+06	0.005	0.032	Corn&Rice

385.22314	$C_{20}H_{34}O_7$	2.3E+07	-0.007	0.005	
385.23840	$C_{24}H_{34}O_4$	9.2E+06	0.005	-0.010	
385.24560	$C_{18}H_{34}N_4O_5$	4.8E+06	-0.011	0.009	Corn
385.25953	$C_{21}H_{38}O_6$	7.5E+06	0.021	0.009	Rice
385.29592	$C_{22}H_{42}O_5$	8.0E+06	0.015	0.001	
385.33232	$C_{23}H_{46}O_4$	6.4E+06	0.009	0.006	

Supplementary Table S7. Identification of compounds characteristic for wheat, corn and rice based on UPLC-ToF-MS fragmentation spectra.

m/z $[M-H]^-$	RT	Starch source	Identification: potential structure [molecular formula]	Identification: Level[1]	MS^2-fragm. (neg.) [x/y][1]	MS^2-fragm. (pos.) [x/y][1]	Literature
164.0348	3.79	wheat	MBOA [$C_8H_7NO_3$]	2	121(18), 149(100), 164(25) [2/2]	67(26), 95(40), 106(19), 107(26), 110(64), 122(17), 151(31), 166(100) [8/8]	[2]
326.0886	3.00	wheat	HBOA-Glc [$C_{14}H_{17}NO_8$]	2	108(47), 149(17), 164(100), 236(61) [3/3]	-	[2-4]
342.0831	3.03	wheat	DIBOA-Glc [$C_{14}H_{17}NO_9$]	2	134(100), 162(11), 180(9), 342(22) [3/3]	-	[3,4]
356.0991	3.14	wheat	HMBOA-Glc [$C_{15}H_{19}NO_9$]	2	123(10), 138(26), 166(9), 194(100), 356(32) [4/4]	-	[3,4]
421.2710	4.40	wheat	N-Acylglutamine [$C_{23}H_{38}N_2O_5$]	3	243(10), 357(100), 375(8) [3/3]	-	*in silico*[5,6]
423.2867	4.62	wheat	N-Acylglutamine [$C_{23}H_{40}N_2O_5$]	3	157(5), 209(27), 221(9), 245(4), 359(100), 377(15), 423(14) [6/6]	-	*in silico*
431.2916	4.49	wheat	N-Acylglutamine [$C_{25}H_{40}N_2O_4$]	3	209(17), 221(16), 245(36), 359(56), 431(100) [4/4]	-	*in silico*
437.2653	4.32	wheat	N-Acylglutamine [$C_{23}H_{38}N_2O_6$]	3	243(20), 373(100), 391(42), 437(9) [NA]	120(29), 121(100), 133(79), 439(62) [NA]	*in silico*
439.2820	4.50	wheat	N-Acylglutamine [$C_{23}H_{40}N_2O_6$]	3	164(4), 173(10), 225(11), 237(4), 245(8), 375(100), 393(42), 439(11) [5/7]	-	*in silico*
206.0458	3.02	corn	Hydroxyoxindol-acetic acid [$C_{10}H_9NO_4$]	2	147(11), 162(100), 188(17), 206(36) [3/3]	85(21), 89(20), 116(52), 144(51), 162(100), 208(25) [5/5]	*in silico*, [7]
337.2382	6.04	corn	Lipid (DiHEtrE) [$C_{20}H_{34}O_4$]	3	169(3), 183(4), 197(4), 211(7), 225(7), 239(16), 276(4), 319(14), 337(100) [8/8]	79(22), 81(65), 93(31), 95(100), 105(34), 107(23), 109(54), 123(31), 135(25),	*in silico*, [8,9]
337.2381	6.09	corn	Lipid (DiHEtrE) [$C_{20}H_{34}O_4$]	3	169(3), 183(3), 197(3), 211(8), 225(7), 239(18), 276(3), 319(12), 337(100) [8/8]		*in silico*
351.2182	6.57	corn	Lipid (TriHETE) [$C_{20}H_{32}O_5$]	3	181(3), 209(6), 211(5), 235(10), 239(13), 253(12), 264(3), 289(3), 305(3), 307(7), 333(6), 351(100)	91(25), 95(26), 113(546), 163(43), 181(54), 241(66), 255(24), 317(34), 335(100),	*in silico* [8,9]
351.2182	6.62	corn	Lipid (TriHETE) [$C_{20}H_{32}O_5$]	3	181(3), 209(6), 211(4), 235(9), 239(11), 253(11), 264(3), 289(3), 305(3), 307(7), 333(5), 351(100)		*in silico*
351.2538	6.34	corn	Lipid [$C_{21}H_{36}O_4$]	3	183(4), 211(5), 225(3), 239(11), 253(9), 290(4), 333(17), 351(100) [NA]	107(27), 279(35), 335(100), 353(31) [NA]	*in silico*
351.2544	6.26	corn	Lipid [$C_{21}H_{36}O_4$]	3	183(4), 211(6), 225(3), 235(2), 239(11), 253(9), 290(5), 333(16), 351(100) [NA]		*in silico*
365.2332	7.17	corn	Lipid [$C_{21}H_{34}O_5$]	3	183(2), 211(2), 223(3), 239(6), 249(4), 250(5), 253(6), 267(5), 294(3), 319(2), 321(4), 347(11),	127(37), 195(68), 255(72), 265(29), 269(30), 349(100), 367(25) [NA]	*in silico*
365.2334	6.87	corn	Lipid [$C_{21}H_{34}O_5$]	3	195(2), 211(4), 223(6), 239(10), 249(11), 253(11), 267(12), 319(3), 321(7), 347(6), 365(100)		*in silico*
365.2337	6.80	corn	Lipid [$C_{21}H_{34}O_5$]	3	224(4), 240(5), 250(9), 251(5), 253(7), 267(3), 268(7), 322(6), 348(5), 365(100) [NA]		*in silico*
237.0768	3.39	corn grits	Coumaroyl glycerol [$C_{12}H_{14}O_5$]	2	117(100), 119(38), 145(76), 163(11), 237(71) [4/4]	65 (13), 91(54), 119(37), 147(100), 239(13) [4/4]	*in silico*, [10]
253.0718	3.15	corn grits	Caffeoyl glycerol [$C_{12}H_{14}O_6$]	2	105(10), 133(87), 135(46), 161(82), 179(12), 253(100) [5/5]	63(11), 77(8), 89(80), 107(4), 117(31), 135(23), 145(25), 163(100), 255(15) [7/8]	*in silico*, [10]
451.1380	2.95	rice	N-Glc-IAA-Asp [$C_{20}H_{24}N_2O_{10}$]	1	132(35), 173(43), 292(15), 335(18), 433(7), 451(100) [5/5]	130(100), 134(14), 147(18), 172(17), 291(63), 453(23) [5/5]	*Co-Chrom*
539.2845	3.50	rice	[$C_{24}H_{40}N_6O_8$]	-	127(3), 167(7), 215(12), 255(10), 424(2), 539(100) [NA]	-	-
542.2621	3.88	rice	Peptide (Glu-Trp-Leu/Ile-Pro) [$C_{27}H_{37}N_5O_7$]	3	127(3), 171(3), 222(7), 240(13), 542(100) [4/4]	116(46), 120(93), 144(32), 159(28), 173(98), 284(34), 316(35), 320(48), 429(47), 544(100) [9/9]	*in silico*
544.2257	2.96	rice	[$C_{22}H_{35}N_5O_{11}$]	-	128(21), 185(6), 215(7), 241(6), 544(100) [NA]	-	-
599.2831	3.77	rice	Peptide (Asp-Ser-Val-Leu-Trp, cyclic) [$C_{29}H_{40}N_6O_8$]	3	240(6), 369(3), 387(28), 599(100) [3/3]	217(11), 227(11), 245(13), 339(15), 340(11), 486(34), 601(100) [6/6]	*in silico*

Supplementary Table S8. Presence of the compounds characteristic for wheat, corn and rice in respective grain foodstuff.

m/z [M-H]$^-$	RT		CG[1]	CS	CF	CO	WG	WS	WF	HWF	RG	RS	RF
164.0348	3.79	MBOA [$C_8H_7NO_3$]	-	-	-	-	-	-	-	**pos.**	-	-	-
326.0886	3.00	HBOA-Glc [$C_{14}H_{17}NO_8$]	-	-	-	-	-	-	**neg.**	**neg.**	-	-	-
342.0831	3.03	DIBOA-Glc [$C_{14}H_{17}NO_9$]	-	-	-	-	**neg.**	-	-	-	-	-	-
356.0991	3.14	HMBOA-Glc [$C_{15}H_{19}NO_9$]	-	-	-	-	-	-	**neg.**	**neg.**	-	-	-
421.2710	4.40	N-Acylglutamine [$C_{23}H_{38}N_2O_5$]	-	-	-	-	-	-	-	-	-	-	-
423.2867	4.62	N-Acylglutamine [$C_{23}H_{40}N_2O_5$]	-	-	-	-	**neg.**	-	**neg.**	**neg.**	-	-	-
431.2916	4.49	N-Acylglutamine [$C_{25}H_{40}N_2O_4$]	-	-	-	-	**neg.**	-	-	-	-	-	-
437.2653	4.32	N-Acylglutamine [$C_{23}H_{38}N_2O_6$]	-	-	-	-	**neg.** **pos.**	-	**pos.**	**pos.**	-	-	-
439.2820	4.50	N-Acylglutamine [$C_{23}H_{40}N_2O_6$]	-	-	-	-	**neg**.	-	-	-	-	-	-
206.0458	3.02	Hydroxyoxindol-acetic acid [$C_{10}H_9NO_4$]	**neg**.[2] **pos**.	**neg**.	**neg**.	-	-	-	-	-	-	-	-
337.2382	6.04	Lipid (DiHEtrE) [$C_{20}H_{34}O_4$]	-	-	-	**neg**.	-	-	-	-	-	-	-
337.2381	6.09	Lipid (DiHEtrE) [$C_{20}H_{34}O_4$]	-	-	-	**neg**.	-	-	-	-	-	-	-
351.2182	6.57	Lipid (TriHETE) [$C_{20}H_{32}O_5$]	-	-	-	-	-	-	-	-	-	-	-
351.2182	6.62	Lipid (TriHETE) [$C_{20}H_{32}O_5$]	-	-	-	-	-	-	-	-	-	-	-
351.2538	6.34	Lipid [$C_{21}H_{36}O_4$]	-	-	-	**neg**.	-	-	-	-	-	-	-
351.2544	6.26	Lipid [$C_{21}H_{36}O_4$]	-	-	-	**neg**.	-	-	-	-	-	-	-
365.2332	7.17	Lipid [$C_{21}H_{34}O_5$]	-	-	-	-	-	-	-	-	-	-	-
365.2334	6.87	Lipid [$C_{21}H_{34}O_5$]	-	-	-	-	-	-	-	-	-	-	-
365.2337	6.80	Lipid [$C_{21}H_{34}O_5$]	-	-	-	-	-	-	-	-	-	-	-
451.1380	2.95	N-Glc-IAA-Asp [$C_{20}H_{24}N_2O_{10}$]	-	-	-	-	-	-	-	-	**neg.**	**neg.** **pos.**	**neg.**
539.2845	3.50	[$C_{24}H_{40}N_6O_8$]	-	-	-	-	-	-	-	-	-	-	**neg.**
542.2621	3.88	Glu-Trp-Leu/Ile-Pro [$C_{27}H_{37}N_5O_7$]	-	-	-	-	-	-	-	-	-	**neg.**	**neg.**
544.2257	2.96	[$C_{22}H_{35}N_5O_{11}$]	-	-	-	-	-	-	-	-	-	**neg.**	-
599.2831	3.77	Asp-Ser-Val-Leu-Trp (cyclic) [$C_{29}H_{40}N_6O_8$]	-	-	-	-	-	-	-	-	-	-	-

[1] CG: corn grits, CS: corn starch, CF: corn flour, CO: corn oil, WG: wheat grits, WS: wheat starch, WF: wheat flour, HWF: whole wheat flour, RG: rice grits, RS: rice starch, RF: rice flour

[2] the respective compound was found in negative (neg.) and/or positive (pos.) ionization mode.

Supplementary figures

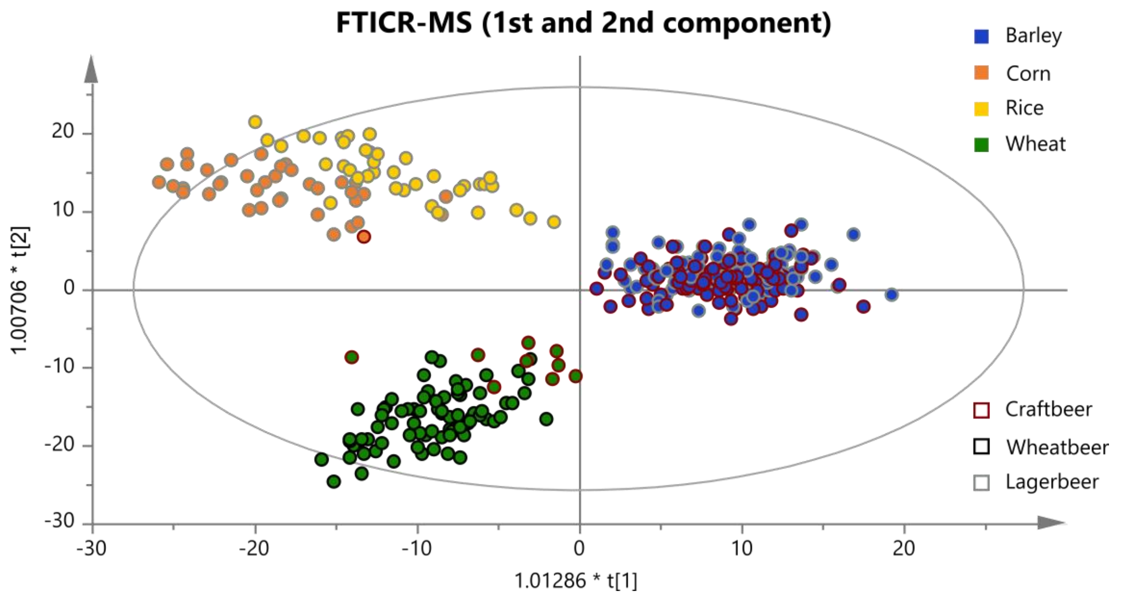

Supplementary Figure S1. Score plot of FTICR-MS data (1st and 2nd component) shows the overlap of barley beers (carbohydrate source) with craft beers (beer type). All measured samples are colored according to their carbohydrate source. The color of the frame is defined by the beer type. Most of the craft beers are brewed with barley as only carbohydrate source.

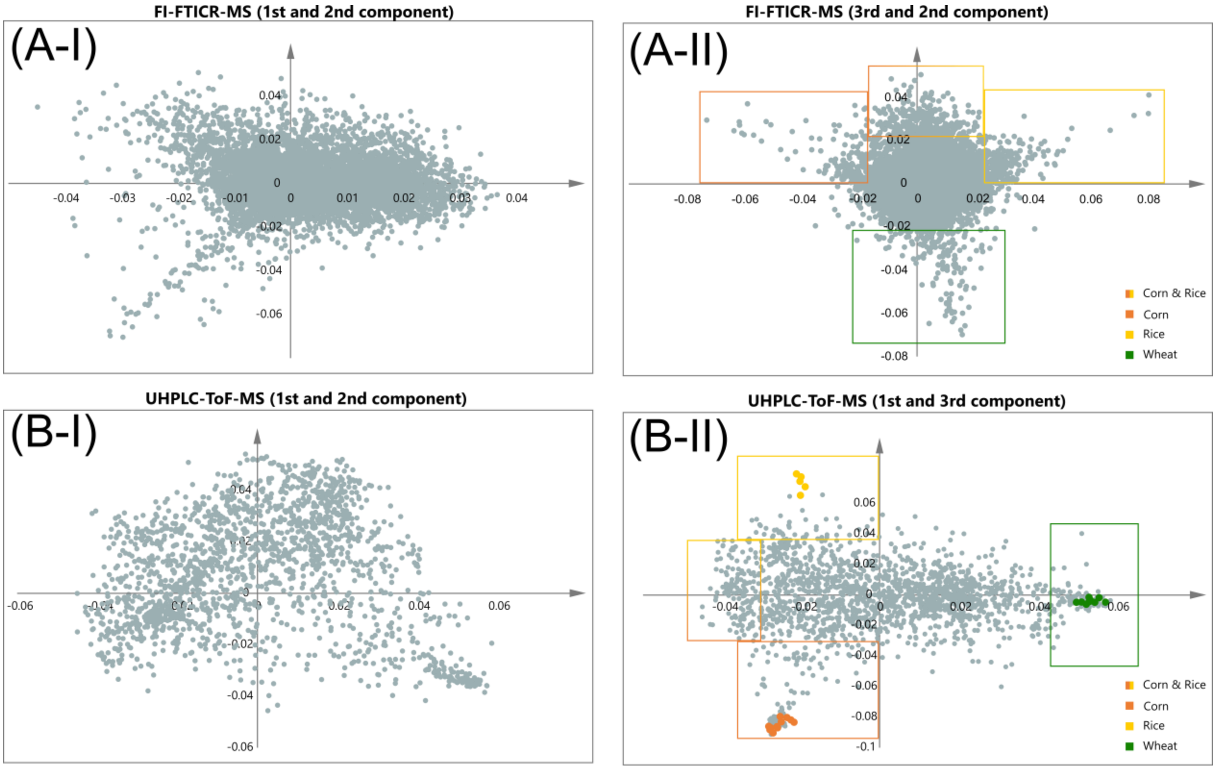

Supplementary Figure 2. Loadings plots of the OPLS-DA of the FI-FTICR-MS **(A)** and UPLC-ToF-MS **(B)** data differentiating the carbohydrate sources used. The position of mass features (grey) indicate their separation significance regarding the beer characteristics given in the score plot (Figure 2). The most significant marker compositions are highlighted in the corresponding color. The first and second components are shown in **(A-I)** and **(B-I)**. The third against the second and the first against the third component are shown in **(A-II)** and **(B-II)** respectively. Colored dots in **(B-II)** could be identified (level 1-3).

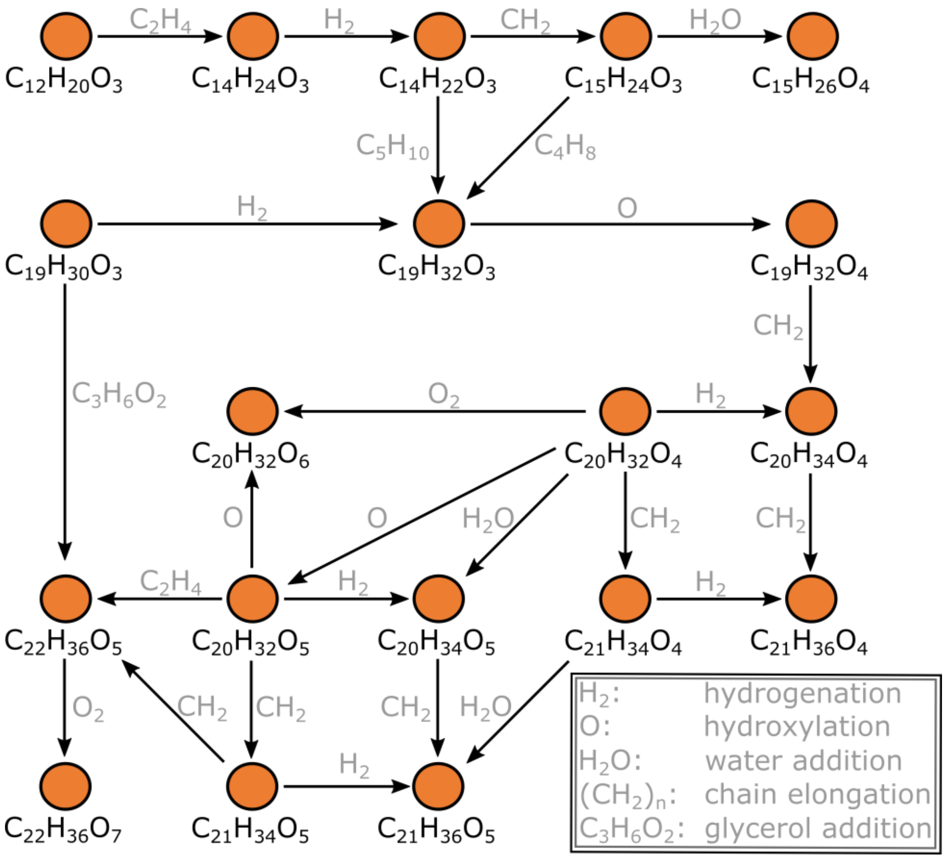

Supplementary Figure S3. Mass difference network excerpt of lipids characteristic for corn. The nodes representing annotations are connected by edges representing potential biochemical reactions. Some connections are neglected for reasons of clarity.

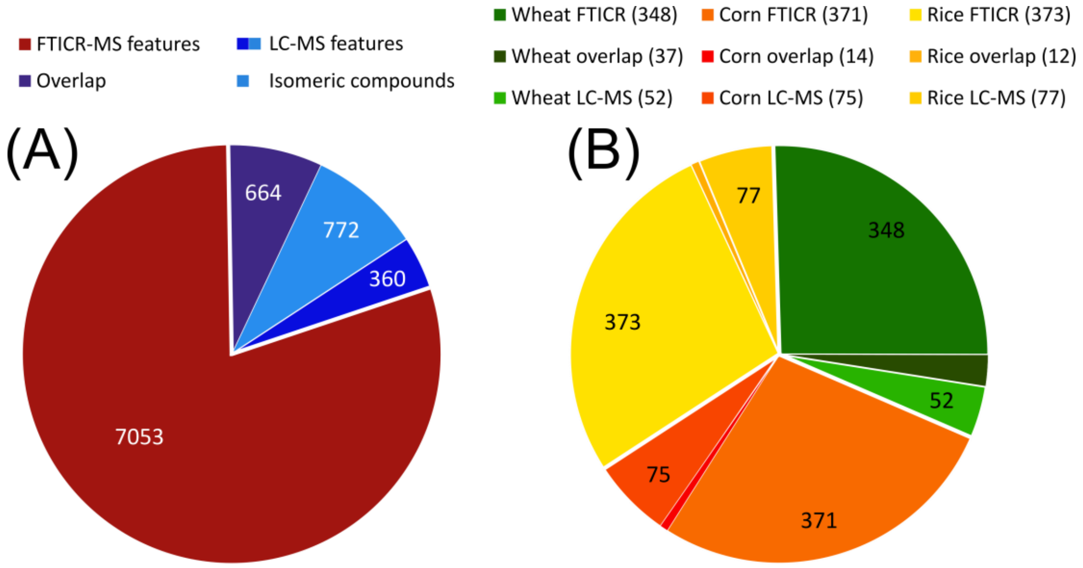

Supplementary Figure S4. Overlap of mass features found in RP-UPLC-ToF-MS (LC-MS) and FI-FITCR-MS (FTICR) within a mass tolerance of ±10 ppm with regard to overall peaks **(A)** and peaks found as potential markers for carbohydrate sources **(B)**. The analytical approaches are differentiated by color in **(A)**. The colors in **(B)** are based on the different carbohydrate sources.

REFERENCES

1 Sumner, L. W. *et al.* Proposed minimum reporting standards for chemical analysis. *Metabolomics* **3**, 211-221 (2007).

2 Bonnington, L. S., Cercelo, D. & Knepper, T. P. Utilisation of electrospray time-of-flight mass spectrometry for solving complex fragmentation patterns: application to benzoxazinone derivatives. *J. Mass Spectrom.* **38**, 1054-1066 (2003).

3 de Bruijn, W. J. C., Vincken, J.-P., Duran, K. & Gruppen, H. Mass spectrometric characterization of benzoxazinoid glycosides from rhizopus-elicited wheat (*Triticum aestivum*) seedlings. *J. Agric. Food Chem.* **64**, 6267-6276 (2016).

4 Pieczonka, S. A., Lucio, M., Rychlik, M. & Schmitt-Kopplin, P. Decomposing the molecular complexity of brewing. *NPJ Sci. Food* **4**, 1-10 (2020).

5 Lai, Z. *et al.* Identifying metabolites by integrating metabolome databases with mass spectrometry cheminformatics. *Nat. Methods* **15**, 53-56 (2018).

6 Ruttkies, C., Schymanski, E. L., Wolf, S., Hollender, J. & Neumann, S. MetFrag relaunched: incorporating strategies beyond in silico fragmentation. *J. Cheminform.* **8** (2016).

7 Fenz, R. *Entwicklung einer HPLC-Methode zum Maisnachweis in Bier* Dr. rer. nat. thesis, Technische Universität zu Braunschweig, (1991).

8 Ferreiros, N. *et al.* Lipoxin A4: Problems with its determination using reversed phase chromatography–tandem mass spectrometry and confirmation with chiral chromatography. *Talanta* **127**, 82-87 (2014).

9 Wheelan, P., Zirrolli, J. A. & Murphy, R. C. Electrospray Ionization and Low Energy Tandem Mass Spectrometry of Polyhydroxy Unsaturated Fatty Acids. *J. Am. Soc. Mass. Spectrom.* **7**, 140-149 (1996).

10 Fenz, R., Ernst, L. & Galensa, R. Phenolcarbonsäuren und ihre Glycerinester in Maisgrits. *Z. Lebensm. Unters. Forsch.* **194**, 252-258 (1992).
